# Study on the interaction preference between CYCD subclass and CDK family members at the poplar genome level

**DOI:** 10.1038/s41598-022-20800-9

**Published:** 2022-10-07

**Authors:** Chengcheng He, Jinghui Liang, Zhaoqun Wu, Xianglin Zhuge, Nan Xu, Hailing Yang

**Affiliations:** National Engineering Research Center of Tree Breeding and Ecological Restoration, Institute of Tree Development and Genome Editing, College of Biological Sciences and Biotechnology, Beijing Forestry University, Beijing, 100083 China

**Keywords:** Evolution, Plant sciences

## Abstract

Cyclin-dependent kinases (CDKs) control the progression of the cell cycle. D-type cyclin (CYCD) is generally believed to form a complex with CDK and control the G1/S transition. In plants, *CYCD* and *CDK* gene families can be divided into 6 (D1–D7) and 7 (CDKA–CDKG) subclasses, respectively. Different subclasses in the *CYCD* and *CDK* families have different numbers, structures and functions. In some heterologous woody plants, the functions of these subclass family members remain unclear. In this study, 43 *CYCD* and 27 *CDK* gene family members were identified in the allodiploid *Populus tomentosa Carr*. Phylogenetic analysis suggested that these *CYCDs* and *CDKs* were divided into 6 and 7 subclasses, respectively, which were the same as other species. The analysis of protein properties, gene structure, motifs, domains, cis-acting elements and tissue-specific expression of all members of these *CYCDs* and *CDKs* showed that the differences between members of different subclasses varied widely, but members of the same subclass especially in the *CDK* gene family were very similar. These findings also demonstrated a strong correlation between *CYCD* and *CDK* gene family members in response to hormones and specific expression. The collinear analysis of *P. tomentosa*, *Populus trichocarpa* and *Arabidopsis thaliana* showed that the expansion patterns of *CYCD* and *CDK* gene families were predominantly whole genome duplications (WGD). The protein interaction prediction results of different subclasses of CYCD and CDKs showed that the interaction between different subclasses of CYCD and CDKs was significantly different. Our previous study found that transgenic *PtoCYCD2;1* and *PtoCYCD3;3* poplars exhibited opposite phenotypes. Y2H and BIFC results showed that the interaction between PtoCYCD2;1 and PtoCYCD3;3 was significantly different with CDKs. This finding might suggest that the functional differences of different CYCD subclasses in plant growth and development were closely related to the different interactions between CYCD and CDK. Our results provide a good idea and direction for the functional study of CYCD and CDK proteins in woody plants.

## Introduction

Cell cycle is a crucial biological process for plant growth and development. In contrast to animal development, plant development is largely post-embryonic. Many organs, such as roots, stems, leaves, and flowers, are inseparable from cell division and differentiation^1–3^. Plant cells are totipotent, and highly differentiated plant cells can still develop into a complete plant^4^. Therefore, the research on the regulation mechanism of plant cell cycle has always been a research hotspot. Cyclins are cyclically synthesised and degraded proteins that interact with CDKs to control the progression of the cell cycle^1,5^. Cyclin-dependent kinase inhibitors (CKIs) inhibit the activity of CDKs by binding to CDKs^6^. Different CDK–cyclin complexes phosphorylate a series of substrates at the key G1-to-S and G2-to-M transition points, thereby triggering the onsets of DNA replication and mitosis, respectively^1^. D-type cyclins (CYCD) are thought to be involved in controlling the G1-to-S transition^7^. When stimulated by cell division signals, CYCD and CDK form an inactive complex, which can be activated by CDK-activating kinase (CAK) phosphorylation^8^. TThe phosphorylation-activated CYCD and CDK complexes can phosphorylate the retinoblastoma-related protein(RBR) protein and release the E2Fa/b-DP transcriptional activator to stimulate the expression of the downstream S phase related genes, which promotes cell entry into the S phase.

Plant CDKs have been classified into seven types, namely, CDKA–CDKG^9^. Different subclasses of CDK proteins have evident differences in structures and functions in the process of plant growth and development. CDKA contains a conserved PSTAIRE motif and plays a pivotal role at the G1-to-S and G2-to-M transition points^1^. Homozygous cdka;1 mutants could be distinguished by the lack of root growth, strongly reduced cotyledon expansion and hypocotyl elongation, minute rosette leaves, and the formation of completely sterile flowers^10^. CDKB and CDKA are most closely related in the evolutionary tree and CDKB contains the PPTALRE or PPTTLRE motif^9,11,12^. CDKB is thought to regulate the G2-to-M transition^1^. *AtCDKB1;1* was highly expressed in guard cells and stomatal precursor cells of cotyledons, suggesting a prominent role for B-type CDKs in stomatal development ^13^. CDKC, containing the PITAIRE motif, can form complexes with CYCT which play a presumed role in transcription elongation by phosphorylating the CTD of RNA polymerase II^1,9,14–16^. Another study found a crucial role of CDKC;2 in modulating cell division and drought response in *Arabidopsis*^17^. The phosphorylation of Thr160 (or the equivalent residue) of CDKs performed by CAKs induces a conformational change that allows proper recognition of substrates. *Arabidopsis* contains two classes of CAK-related genes (i.e. *CDKD* and *CDKF*)^1,18,19^. Among these genes, CDKD can form a complex with CYCH, which is phosphorylated by CDKF to perform CAK enzyme activity^1,20^. *Atcdkf;1* mutant showed defects in cell division and cell expansion and caused stunted growth of shoots and roots^21^. The *Atcdkd;1 Atcdkd;3* double mutant was gametophytic-lethal, suggesting that CDKD was irreplaceable in the gametogenesis and embryogenesis processes^22^. CDKE is a class of proteins containing the SPTAIRE motif^9^. AtCDKE;1, also known as HUA ENHANCER3, was found to play an important role in floral organ and cell expansion in leaves^23^. CDKG proteins contain the PLTSLRE motif. AtCDKG1 could form complexes with CYCL and was essential for synapsis and recombination during male meiosis^24^. CDKG1 and CDKG2 have been shown to mediate alternative splicing (AS) of downstream genes. CDKG1 regulated the AS of the splicing factor U2AF65A in vegetative tissues and CalS5 mRNA in pollen^25,26^.

In *Arabidopsis*, 10 *CYCD* genes had been identified and classified into six subclasses, i.e. *CYCD1*, *CYCD2/4*, *CYCD3*, *CYCD5*, *CYCD6* and *CYCD*7^1,27,28^. Fourteen *CYCD* genes were found in rice (*Oryza sativa L.*)^29^. Sixteen *CYCD* genes were found in tomato^30^. A total of 24 *CYCD* genes were discovered in *Populus trichocarpa*^12^. A total of 17, 40, and 18 *CYCD* genes were found in *Medicago truncatula*, soybean (*G. max*) and common bean (*P. vulgaris*), respectively^31^. The number of CYCDs varies greatly in different species, which may imply that CYCD is very related to the evolution and environmental adaptation of species. Plant cyclins have a conserved region of 250 amino acids, called the cyclin core box, which is composed of Cyclin_N and Cyclin_C domains^32^. Most CYCDs sequences have the conserved AA sequence motif LxCxE (x represents any amino acids) at the N-terminus, which is essential for RBR binding^33^. The PEST region, which is rich in proline (P), glutamic acid (E), serine (S), and threonine (T) residues, is a marker for unstable proteins^34^. Studies showed that CYCDs played an important role in cell proliferation. Loss-of-function mutations in *cycd1;1* and *cycd4;1* genes led to delayed cell proliferation in the *Arabidopsis* root apical meristem, ultimately resulting in few cells in the division phase and slow radicle elongation in *Arabidopsis* seedlings^35^. The deletion of the *cycd4;1* gene reduced lateral root density in *Arabidopsis*, and the treatment with auxin increased lateral root density^36^. The *AtCYCD2;1* gene over expression stimulated the proliferation of lateral root cells, leading to changes in sensitivity to auxin of columella cells to auxin^37^. The three *CYCD3* genes of *Arabidopsis* were all expressed to be related to the secondary growth of plants. Amongst them, the secondary growth of the *cycd3;1* mutant plant was evidently blocked, and the stem thickness was reduced^38^. The activation of CYCD7 in the central cell and early endosperm promoted early endosperm and embryo development^39^.

Populus (poplar) species are important model organisms for molecular genetics studies of woody plants^40,41^. *Populus tomentosa*, also known as Chinese poplar, is widely cultivated in China which is an important tree species in forest production and forest reclamation projects along the Yellow River. Most studies on CYCDs and CDKs focus on the function of a single CYCD or CDK protein by their influence on plant growth and development. There are few studies on the mechanism of CYCD-CDK complexes on cell proliferation and differentiation especially in perennial woody plants. Preliminary work in our laboratory found that *PtoCYCD2;1* and *PtoCYCD3;3* overexpressed "741" poplar had remarkably different phenotypes^12,42^. This study systematically analysed the number of members, gene structure, protein properties, chromosomal distribution, the expansion patterns and the interaction prediction amongst different subclasses of *P. tomentosa CYCD* and *CDK* gene families. After that, the interaction partners of PtoCYCD2;1 and PtoCYCD3;3 were identified by in vitro (yeast two-hybrid [Y2H] assay) and in vivo (bimolecular fluorescent complimentary [BiFC] assay) experiments. Finally, the phenotypes of *PtoCYCD2;1* and *PtoCYCD3;3* overexpressed “741” poplar were used to analyse the functional differences between D2 and D3 subclasses in plant cell proliferation and differentiation. Our research provides a innovative direction and idea for studying the functions of plant CYCD and CDK proteins.

## Results

### Identification of the CYCD and CDK gene families in *Populus tomentosa*

**Figure 1:**
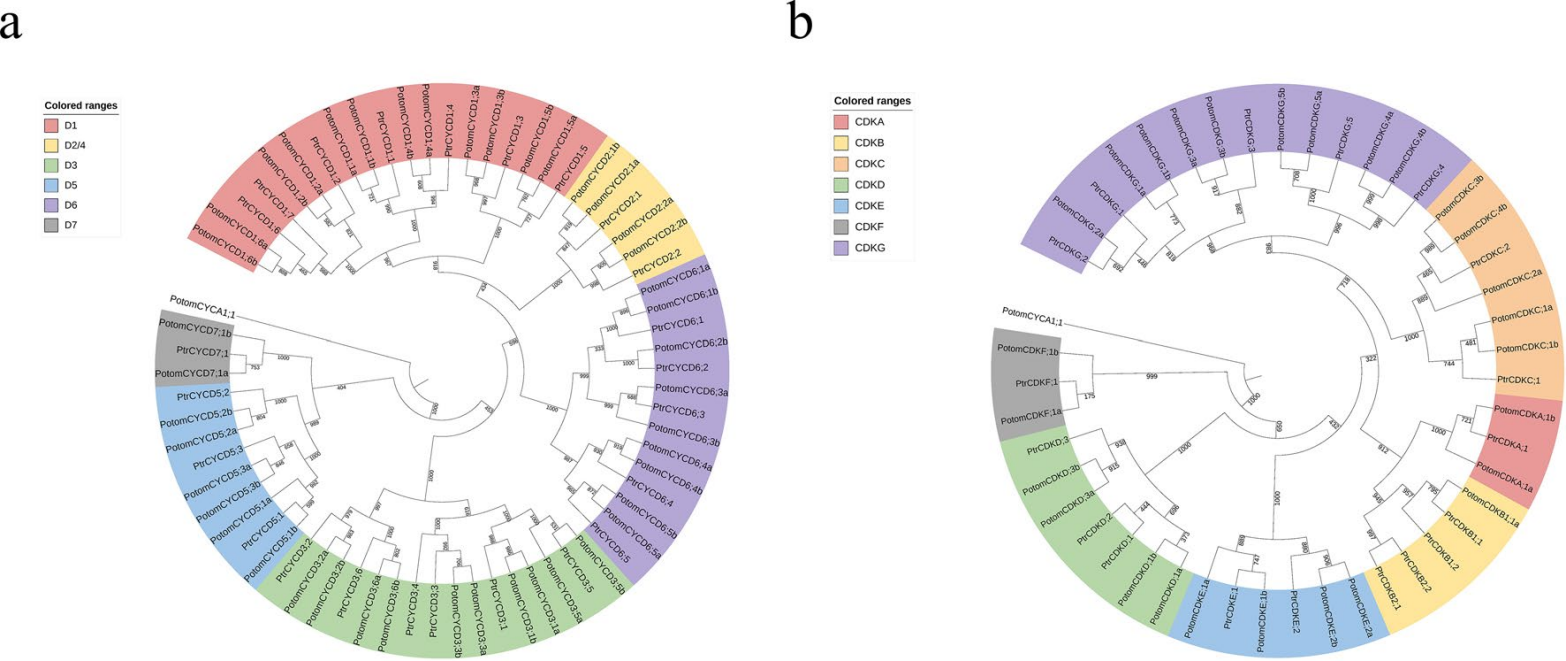
Phylogenetic tree analysis of D-type cyclin (CYCD) and cyclin-dependent kinases (CDK) gene family in *P. tomentosa* and *P. trichocarpa.* A *PotomCYCA* gene from *P. tomentosa* genome was selected as an outgroup gene. (**a**) Phylogenetic tree analysis of 43 PotomCYCDs and 22 PtrCYCDs. All CYCDs were classified into six distinct groups on the basis of the subfamily of *P. trichocarpa* CYCDs (from D1 to D7) and were distinguished by different colours. (**b**) Phylogenetic tree analysis of 27 PotomCDKs and 18 PtrCDKs. All CDKs were classified into seven distinct groups on the basis of the subfamily of *P. trichocarpa* CDKs (from CDKA to CDKG) and were distinguished by different colours.

To identify *CYCD* and *CDK* genes in *P. tomentosa*, hidden Markov models (HMMs) and Blastp were used to query the whole genome. After the elimination of redundant sequences and examination of domains, we finally identified 43 *CYCD* and 27 *CDK* family members (Table S1). A phylogenetic tree was constructed with 24 *CYCD* and 18 *CDK* in *Populus trichocarpa* (*PtrCYCD* and *PtrCDK*) to classify and name the successfully identified members^12^ (Fig. 1). Results showed that 47 *PotomCYCDs* were divided into six subclasses, of which 12, 4, 10, 6, 9 and 2 members belonged to D1, D2/4, D3, D5, D6 and D7 subclasses, respectively. A total of 27 *PotomCDKs* were divided into seven subclasses, which belonged to 2 members of the CDKA subclass, 1 member of the CDKB subclass, 5 members of the CDKC subclass, 4 members of the CDKD subclass, 4 members of the CDKE subclass, 2 members of the CDKF subclass, and 9 members of the CDKG subclass. The identified members of the *P. tomentosa* gene family were named in accordance with the gene family members of *P. trichocarpa*. Considering that *P. tomentosa* is an allodiploid, a was added to the name of the gene searched from subgenome A (PtA), and b was added to the name of the gene searched from subgenome D (PtD). In phylogenetic tree analysis, most of genes had their alleles with close relationships. However, we found *PotomCYCD6;3a* was closer to the orthologs gene *PtrCYCD6;3* rather than its allele *PotomCYCD6;3b*. This phenomenon was also found between *PotomCYCD3;5*, *PotomCYCD5;1*, *PotomCYCD7;1*, *PotomCDKA;1*, *PotomCDKE;1* and *PotomCDKF;1* and there corresponding orthologs, which revealed that these alleles differentiated during the evolutionary process. An interesting finding was that a small number genes *PotomCYCD6;2b*, *PotomCDKG;2a*, *PotomCDKC;2a*, *PotomCDKC3;b*, *PotomCDKC4;b* and *PotomCDKB1;1a* were lack of their alleles. We speculated that it was the chromosomal variation and transposon insertion that resulted in the loss of these alleles. All these evidences indicated that *PotomCYCDs* and *PotomCDKs* were both conserved and differentiated.

To analyse the sequence differences of alleles from different subgenome, we analysed the protein sequence identity of all members of the two gene families. Results showed that in the *PotomCYCD* gene family, PotomCYCD1;3a and PotomCYCD1;3b (99.0%) had the highest sequence similarity, and PotomCYCD1;2a and PotomCYCD1;2b (80.6%) had the lowest sequence similarity (Table S2). In the *PotomCDK* gene family, the highest sequence similarity was PotomCDKE;2a and PotomCDKE;2b (99.5%), and the lowest sequence similarity was PotomCDKA;1a and PotomCDKA;1b (82.4%, Table S3). At the same time, we also investigated the basic characteristics of the two family members, such as their AA length, isoelectric point (PI), molecular weight (MW), and subcellular localization (Table S4). Results showed that the AA length of *PotomCYCD* gene family varied from 256 to 408 AA. The largest protein was PotomCYCD2;1b (45.24 kDa), and the smallest protein was PotomCYCD6;2b (29.51 kDa). The PI of most CYCD proteins varied around 5–7. Subcellular localisation prediction results indicated that all PotomCYCD proteins were located in the nucleus. The difference was that the basic characteristics of different subclasses of proteins in the *PotomCDK* gene family varied remarkably, but the characteristics of different members of the same subclass were relatively similar. The CDKG subclass (except PotomCDKG;2a) had the largest protein (78.65–89.63 kDa), and the CDKA and CDKB subclasses had the smallest protein (33.79–36.53 kDa). Although PotomCDKG;2a is only 117aa in length, it contains part of the conserved domains required by CDK, and it is speculated that it might not be functional due to its short length or mutated during evolution. The protein PIs of different CDK subclasses different but were very similar in the same subclass. Subcellular localisation prediction results showed that all PotomCDK proteins were located in the nucleus and that PotomCDKA;1b might also be located in the cytoplasm. PotomCDKG;4b might also be located in the cell membrane and cytoplasm. PotomCDKG;5a might also be located in the cell membrane.

### CYCD and CDK gene structure and motif analyses

**Figure 2:**
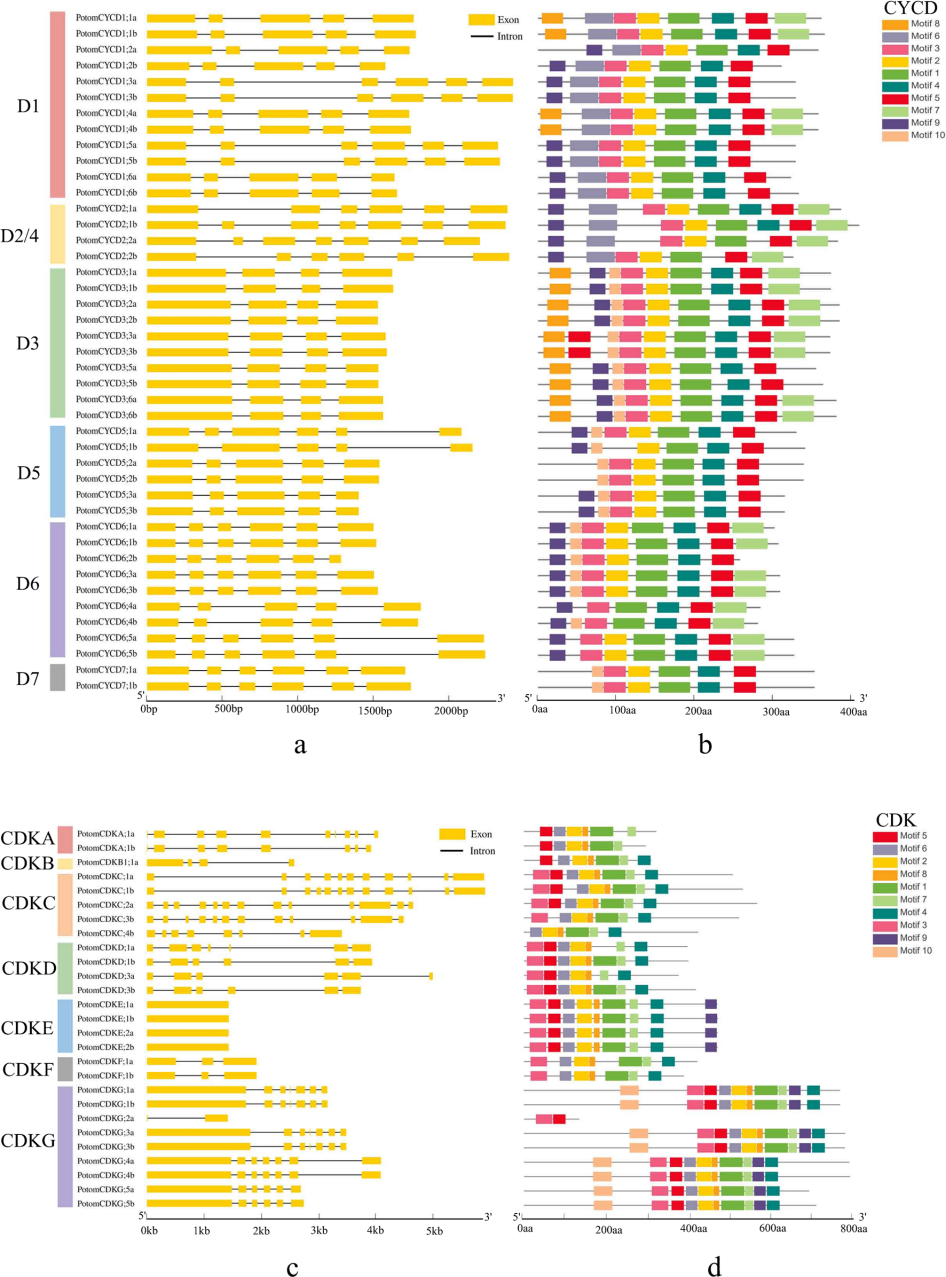
Gene structure and conserved motif compositions of *PotomCYCDs* and *PotomCDKs.* (**a**) Exon/intron structures of *PotomCYCDs*. (**b**) Architecture of conserved protein motifs of PotomCYCDs. (**c**) Exon/intron structures of *PotomCDKs.* Yellow boxes and black lines indicate exons and introns, respectively, at each *CYCD* and *CDK* gene. (**d**) Architectures of conserved protein motifs of PotomCDKs. These coloured boxes indicate distinct motifs and their corresponding positions in each CYCD and CDK protein sequence. The detailed characteristics of each motif are shown in Table S5.

Gene structural diversity and conserved motif divergence are possible mechanisms for the evolution of multigene families^43^. To further study the gene and protein structure of the *CYCD* and *CDK* gene family, we analysed the number and distribution of exons. The results of gene structure analysis showed that the structures of *PotomCYCD* genes were similar and that the number of exons varied from 4 to 7. The number of exons in the D3 subclass was 4, and the number of exons in other subclasses except *PotomCYCD2;1b* and *PotomCYCD2;2a* was 5 or 6 (Fig. 2a). The results of the gene structure analysis of *PotomCDK* genes showed that the number of exons in CDK ranged from 1 to 13. The gene structure amongst members of the same subclass was relatively conserved. For example, none of the four members of CDKE were introns. Amongst the nine members of CDKG, eight members consisted of 1 long exon and 5/6 short exons. A high number of exons were found in CDKA and CDKC, whereas only 3 exons were observed in the CDKF subclass (Fig. 2c).

To elucidate the distribution of the motifs in CYCD and CDK proteins and their function, ten types of motifs and their distributions of CYCDs and CDKs were predicted using the MEME program (Fig. 2). Our results indicated that CYCDs contained similar motif types. However, some differences existed amongst different subclasses. Motifs 1–5 were contained in all subclasses. Motif 6 was unique to D1 and D2/4 subclasses. Motifs 7 and 8 were contained in most subclasses except D5 and D7. Motif 9 was only D7 subclass Class missing. Motif 10 was included in most subclasses except D1 and D2/4 (Fig. 2b). In the CDK gene family, motif 1, 2 and 6–8 were included in all subclasses. Motif 3 was included in most subclasses except CDKA and CDKB. Motif 4 was not found in CDKA subclasses. Motif 5 was not found in CDKF. Motif 9 was only found in CDKE and CDKG subclasses. Motif 10 was unique to CDKG (Fig. 2d). Some interesting findings appeared in some alleles. The *PotomCYCD5;1a* has one more exon than its allele *PotomCYCD5;1b* and motif 3 presented in the former but not the latter. *PotomCYCD6;2b* without allele was lack of motif 7 when comparing to other *PotomCYCD6*. *PotomCDKB1;1a*, the only one member in *PotomCDKB*, had almost the same motifs but an extra motif 4 than the *PotomCDKA;1*. *PotomCDKC;4b* was lack of the motif 3 and motif 5 when comparing to other *PotomCDKC*. These findings revealed that some special alleles might have different gene structures and conserved motifs leading to different functions.

At the same time, we analysed the domains and conserved motifs of CYCDs. LxCxE is a key motif for CYCD binding to RBR^33^. The PEST sequence, a region full of P(Pro), E(Glu), S(Ser) and T(Thr), might result in itself degradation and were often found in D-type cyclins^32–34^. Results showed that all PotomCYCD proteins had Cyclin_N and Cyclin_C domains. No LxCxE motif was observed in the CYCD6 subclass, and the LxCxE motif existed in other subclass proteins. Most CYCD proteins had the PEST motif, but the position of the PEST motif was not fixed (Fig. S1). Multiple sequence alignment with *P. trichocarpa* CDK gene family proteins showed that their conserved domains were highly consistent with their orthologous proteins in *P. trichocarpa* (File S1). Amongst them, CDKA had PSTAIRE, CDKB had PPTALRE or PPTTLRE, CDKC had PITAIRE, CDKE had SPTAIRE, and CDKG had PLTSLRE^9^. However, PotomCDKC;3b and PotomCDKC;4b were not observed with this characteristic motif.

### Prediction analysis of Cis-acting elements within *CYCD* and *CDK* genes

Specific cis-element motifs can be recognised by transcription factors and participate in gene expression regulation. To further study the potential regulatory mechanisms of *PotomCYCDs* and *PotomCDKs* in a diversified biological process, particularly in plant hormones and specific expression, 2.0 kb upstream sequences from the translation start sites of *CYCD* and *CDK* genes were submitted to the PlantCARE database to detect cis-elements. Results showed that the types and numbers of various cis-acting elements of genes in *PotomCYCDs* and *PotomCDKs* were similar, thereby implying their functional relatedness. Multiple hormone-responsive elements, such as ABA responsive (DRE1, ABRE, ABRE2, ABRE3a, ABRE4, TCA-element), auxin and/or salicylic acid activation (as-1), auxin responsive (TGA-element), ethylene responsive (ERE), gibberellin responsive (GARE-motif, P-box, TATC-box) and MeJA responsive (CGTCA-motif, TGACG-motif) elements, were found in cis-acting elements in two gene families. Cis-acting element prediction results showed that the two gene families had similar responses to hormones and had the most responsive elements responsive to ABA followed by ethylene responsive. The numbers and positions of various hormone responsive elements on the promoters of each gene are shown in the Fig. S2–S3. In the *CYCD* gene family, the numbers of ABA responsive and ethylene responsive elements in the D7 subclass with only 2 members were 13 and 10, respectively, but no gibberellin responsive and auxin responsive elements were found in the D7 subclass, which might suggest that the D7 The subclass predominantly responded to ABA and ethylene. The number of MeJA responsive elements in the D5 subclass with 6 members was 18, which was the largest amongst all subclasses. This result might suggest that the D5 subclass predominantly responded to MeJA. In the D6 subclass with only 9 members, 16 Gibberellin responsive elements were found, accounting for the largest proportion and suggesting that the D6 subclass predominantly responded to gibberellin. The auxin responsive element was found in all D1–D5 subclasses but not in D6 and D7 subclasses (Fig. S2). In the *CDK* gene family, the number of ABA and ethylene responsive elements in CDKD subclasses with only 4 members was as high as 20 and 17, but gibberellin responsive and auxin responsive elements were not found in CDKD subclasses, which might imply that CDKD subclasses responded to ABA and ethylene. The numbers of MeJA responsive, gibberellin responsive and auxin responsive elements accounted for 8, 3 and 2, respectively, in CDKF subclasses with only 2 members and had the largest proportion. Auxin responsive element was not found in CDKA, CDKB and CDKD subclasses (Fig. S3).

A number of specific expression elements was also found in cis-acting elements in the two gene families, and the proportions of specific expression elements in the two gene family members were also similar. Pollen specific activation elements were the most numerous, with 49 and 41 in *CYCD* and *CDK* families, respectively. In addition, two families also included some different cis-acting elements, such as the seed specific regulation element (RY-element) that only existed in the *CDK* gene family and the cell cycle regulation element (MSA- like) that only existed in the *CYCD* gene family (Fig. S2–S3).

### Chromosomal distribution and synteny analyses

**Figure 3:**
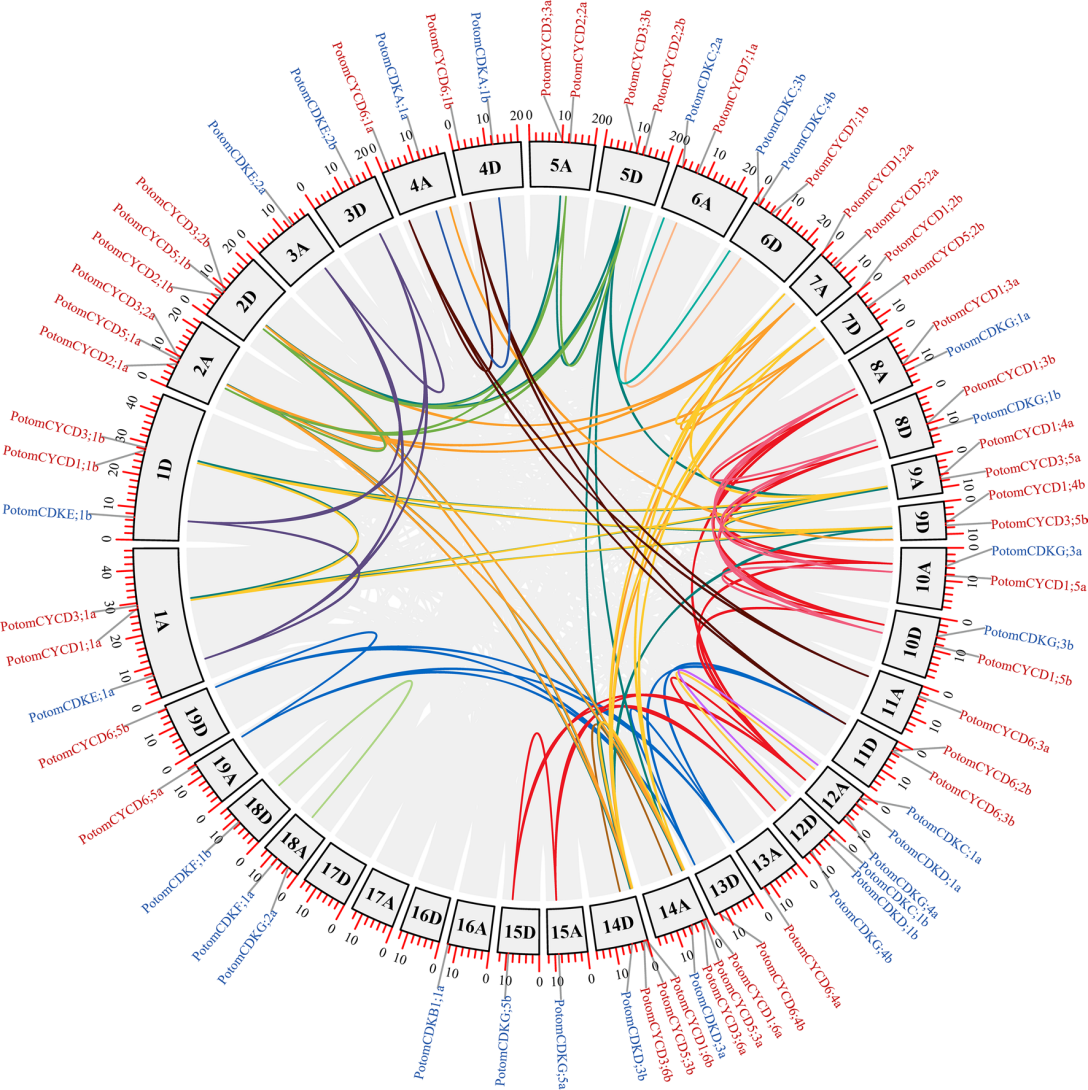
Circos figure for chromosome distribution with synteny links. Grey and colourful lines represent synteny blocks and duplicated *CYCD* and *CDK* gene pairs, respectively, in *P. tomentosa*. The gene ids in red font are members of the *PotomCYCD* gene family, and the gene ids in blue font are members of the *PotomCDK* gene family.

On the basis of the information from the *P. tomentosa* genomic database, we determined the chromosomal distributions of *CYCD* and *CDK* genes (Table S4). Results suggested that *CYCD* genes were distributed on 26 chromosomes, whereas *CDK* genes were mapped onto 21 chromosomes (Fig. 3). Although 35 chromosomes contained *CYCD* or *CDK* genes, the overall distribution was mostly nonuniform. Chromosomes 2A, 2D, 14A and 14D all contained three *CYCD* genes, whereas only one *CYCD* gene was distributed on chromosome 4A. Chromosomes 12A and 12D both contained 3 members of *CDK* genes, whereas chromosome 1A contained only one *CDK* gene. Interestingly, chromosomes 16D, 17A and 17D did not contain any *CYCD* or *CDK* gene.

We constructed a synteny analysis between *CYCD* and *CDK* genes in *P. tomentosa* (Table S6). The synteny blocks and the duplicated *CYCD* and *CDK* gene pairs were showen by the grey and colourful lines (Fig. 3). All *CYCD* genes and 24 of 27 *CDK* genes were identified as collinear genes involving WGD or segmental duplication, whereas 2 *CDK* genes (i.e. *PotomCDKC;4b* and *PotomCDKG;2a*) were considered as dispersed genes, and 1 *CDK* gene (i.e. *PotomCDKB1;1a*) was considered as a singleton gene (Table S6). Remarkably, some *CYCD* and *CDK* genes were associated with at least five syntenic gene pairs, which implied that these genes might be involved in some critical roles during the evolutionary process. Interestingly, tandem duplication events were not found between *CYCD* and *CDK* genes in *P. tomentosa*. Evidence suggested that WGD or segmental duplication led the expansion of *CYCD* and *CDK* genes in *P. tomentosa*.

**Figure 4:**
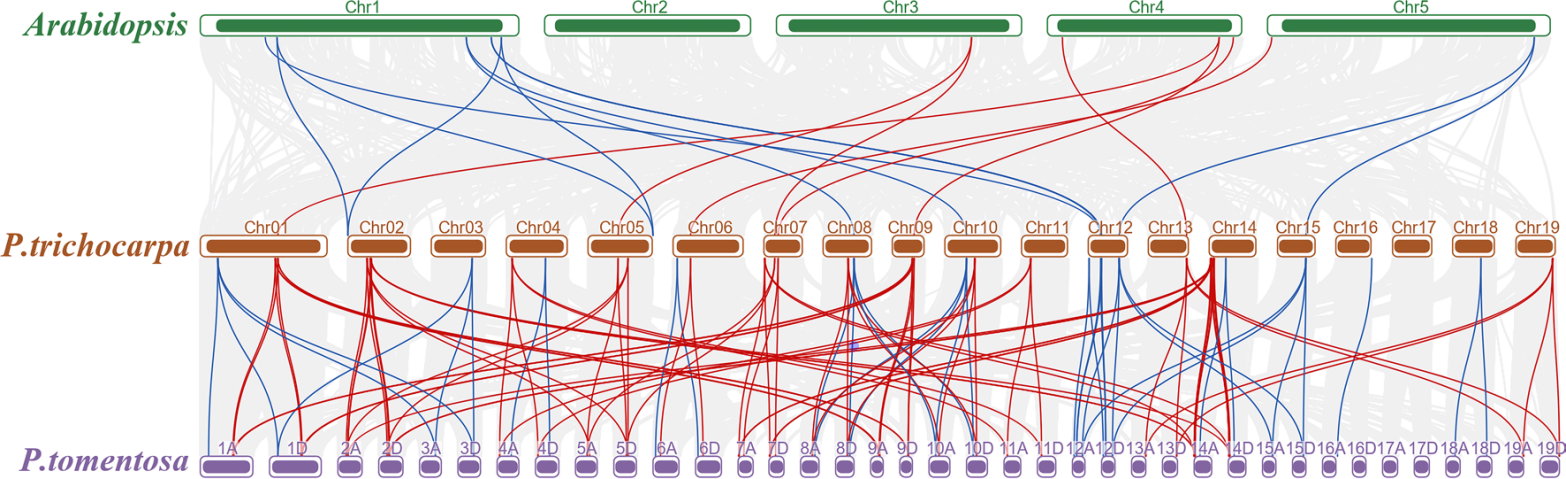
Synteny of *CYCD* and *CDK* genes in *Arabidopsis*, *P. trichocarpa* and *P. tomentosa*. Red and blue lines represent duplicated *CYCD* and *CDK* gene pairs, respectively.

To further explore the evolutionary relationships of the *CYCD* and *CDK* gene families, we performed syntenic analyses amongst *Arabidopsis*, *P. trichocarpa* and *P. tomentosa* (Table S7). Between *Arabidopsis* and *P. trichocarpa*, 7 *CYCD* and 11 *CDK* gene pairs were found. A total of 81 *CYCD* and 38 *CDK* gene pairs were identified between *P. trichocarpa* and *P. tomentosa* (Fig. 4). We found that 4 of 7 *CYCD* gene pairs and 9 of 11 *CDK* gene pairs between *Arabidopsis* and *P. trichocarpa*, respectively, were distributed on chromosome 1 and 4 in *Arabidopsis*. In *P. trichocarpa*, *CYCD* gene pairs were predominantly distributed on chromosomes 1, 2, 7, 9, 14 and 19, whereas *CDK* gene pairs were predominantly located on chromosomes 1, 3, 8, 10 and 12. The situations of *CYCD* and *CDK* gene pair distributions on chromosomes in *P. tomentosa* were similar to *P. trichocarpa*. Between *P. trichocarpa* and *P. tomentosa*, although abundant gene pairs were identified, 1 *CYCD* (i.e. *PotomCYCD6;3b*) and 2 *CDK* (i.e. *PotomCDKC;4b* and *PotomCDKG;2a*) genes did not find any syntenic gene.

### Expression patterns of CYCD and CDK genes in different tissues

To investigate the possible roles of the *PotomCYCDs* and *PotomCDKs*, the expression levels of 43 *CYCD* and 27 *CDK* genes were determined by transcriptome results in three various tissues, i.e. leaf, stem and root^41^. Our results indicated that *CYCD* and *CDK* genes showed similar expression profiles in different tissues. Most genes were expressed in all three tissues, and alleles from different subgenomes had similar or even the same expression pattern (Figs. S4–S5). In *CYCD* and *CDK* gene families, most genes showed this expression trend, with the highest expression in stems, followed by roots and leaves. In the *CYCD* gene family, most D3 subclass gene expression levels conformed to this trend. However, *CYCD3;1a*, *CYCD3;2a* and *CYCD3;2b* gene expression levels were highest in roots, and the expression of *CYCD3;2b* in leaves was highest amongst all genes. In terms of the overall expression levels of genes in different subclasses, D3 subclass genes had the highest expression, followed by the D1 and D2 subclasses, whereas the D7 subclass was not expressed in all tissues. In the *CDK* gene family, the expression trends of CDKA and CDKB subclass genes were the same as those mentioned before. The genes of other subclasses are also highly expressed in leaves and roots, such as *CDKC;1a/b* and *CDKG;1a/b* had the highest expression in leaves and *CDKC;2a/b* and *CDKG;4a/b* had the highest expression in roots. In terms of the overall expression levels of genes in different subclasses, the CDKA subclass had the highest gene expression followed by the CDKG subclass. These results suggested that *CYCD* and *CDK* genes had similar expression patterns, implying their functional relevance, and the overall expression level of *CYCD* genes was lower than that of *CDK* genes. These results might also indicate that alleles with the same function as in an allodiploid species co-regulated the growth and development of the organism.

### Prediction of interaction between CYCD and CDK gene families

**Figure 5:**
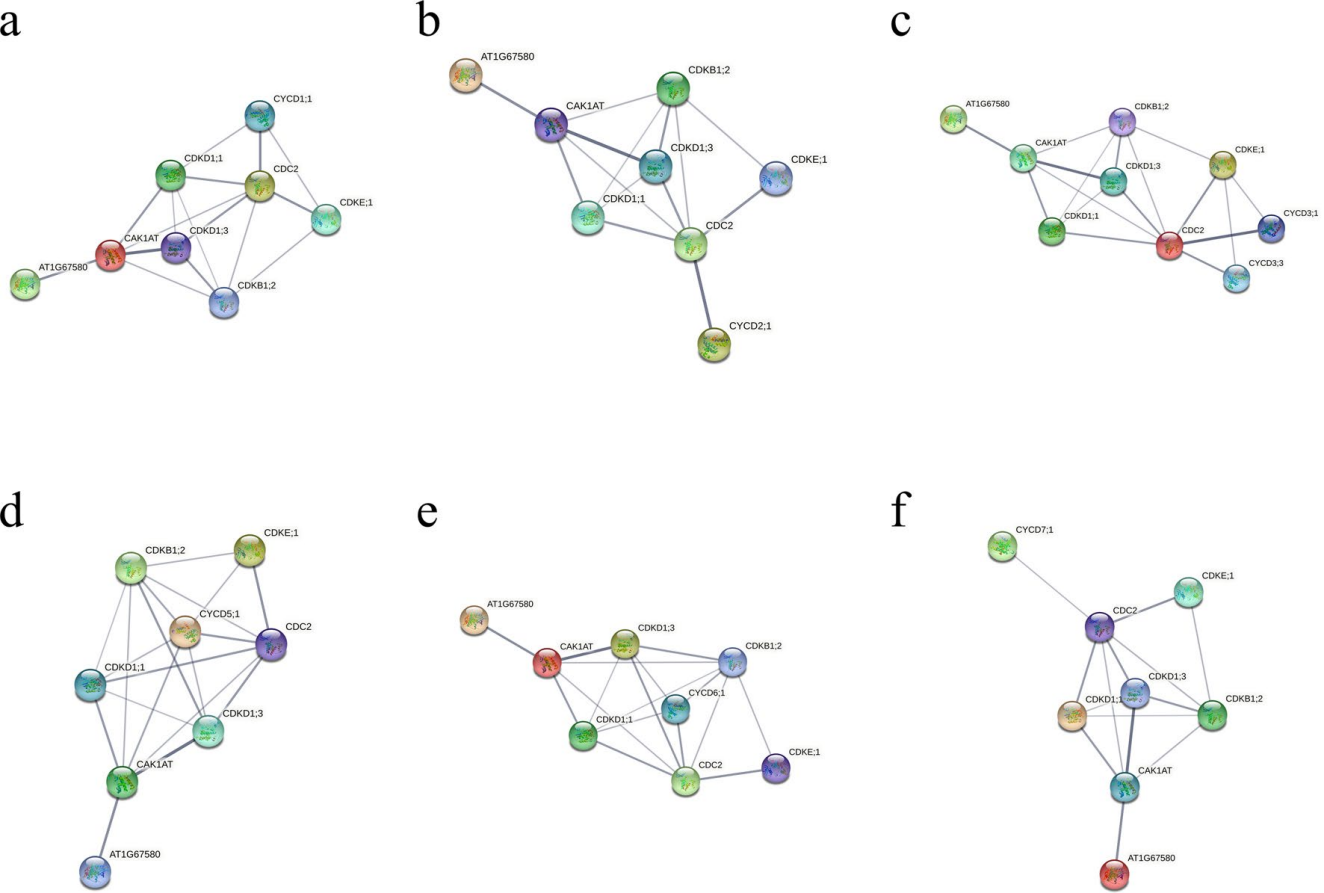
Prediction of the interaction between different subfamilies of *PotomCYCDs* and *PotomCDKs* gene family proteins by using the STRING website. (**a**) Interaction between D1 subfamily and CDKs. (**b**) Interaction between D2/4 subfamily and CDKs. (**c**) Interaction between D3 subfamily and CDKs. (**d**) Interaction between D5 subclass and CDKs. (**e**) Interaction between D6 subfamily and CDKs. (**f**) Interaction between D7 subfamily and CDKs. Line thickness indicates the strength of data support.

In order to analyze the interaction between different CYCD and CDK proteins, the STRING website was used to predict the interaction between different CYCD subclasses and CDK proteins. First, all *PotomCYCD* and *PotomCDK* genes were compared with the *Arabidopsis* database of the STRING website. The comparison results and annotation information are shown in Table S8. The interaction between different subclasses of CYCD and CDK was predicted and the line thickness indicates the strength of data support (Fig. 5). Results showed that CDKA (CDC2) was at the core of the interaction relationship. The protein interaction prediction results showed that the D1 subclass could interact with CDKA, CDKD1;1 and CDKE;1 and had the strongest interaction with CDKA. The D2/4 subclass only could have a strong interaction with CDKA. In the D3 subclass, the proteins CYCD3;1 and CYCD3;3 were obtained from the alignment and could interact with CDC2 and CDKE;1, and the interaction with CDKA was stronger. The D5 subclass could interact with CDKA, CDKB1;2, CDKD1;1, CDKD1;3, CDKE;1 and CAK1AT (CDKF). The D6 subclass could interact with CDKA, CDKB1;2, CDKD1;1 and CDKD1;3 has an interaction relationship. The D7 subclass only had a weak interaction relationship with CDKA (Fig. 5). These results suggested that different subclasses of CYCD proteins might differ in gene sequence and protein properties and in their interaction with CDKs.

### PtoCYCD3;3 interacted with 12 PtoCDK proteins in vivo

**Figure 6:**
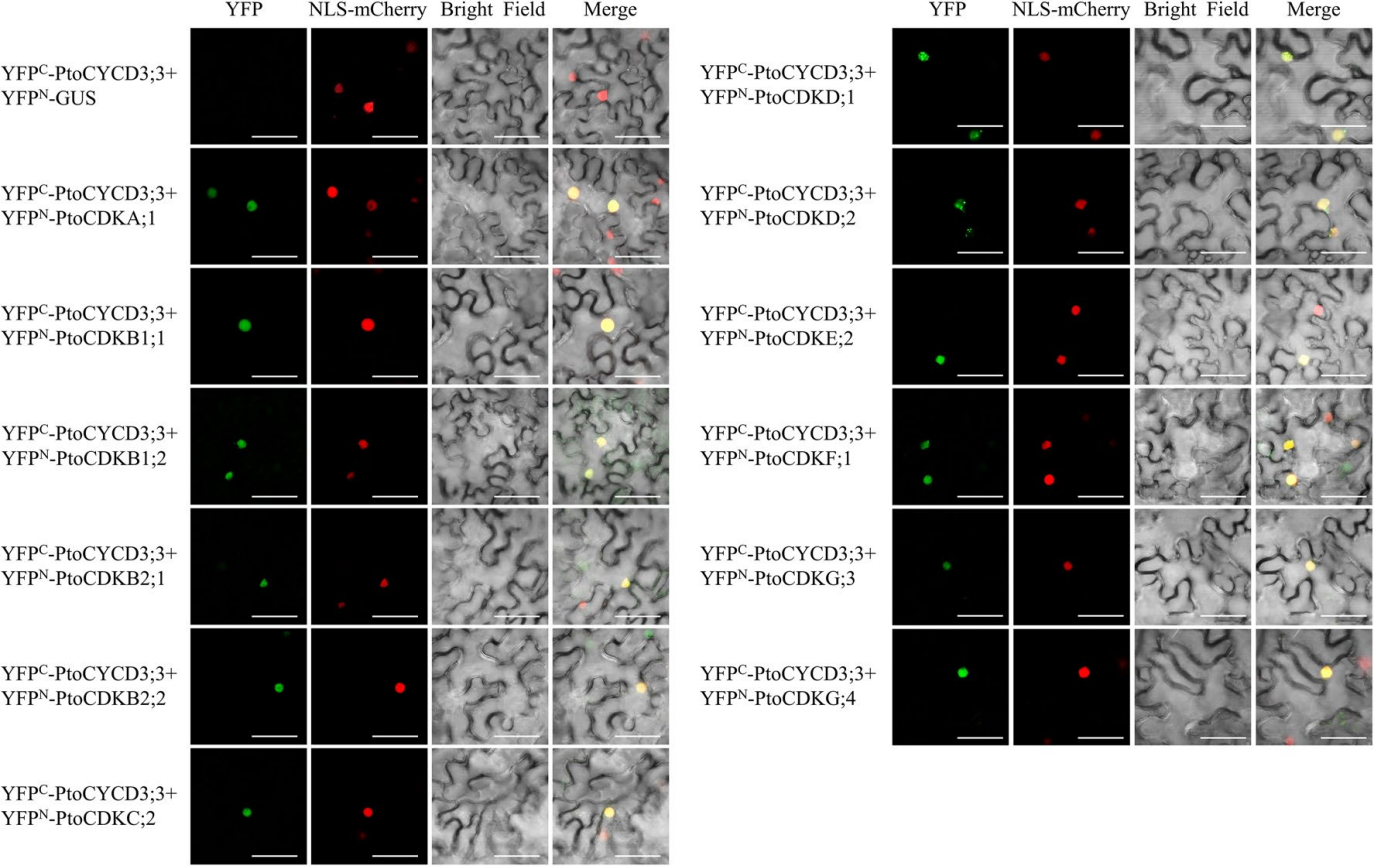
BiFC validation in tobacco epidermis. *PtoCYCD3;3* connects pSPYCE(MR) vector and PtoCDKs connects pSPYNE(R)173 vector. After co-expression in tobacco leaf epidermal cells, the fluorescence signal was observed under a laser confocal microscope. YFP^C^-PtoCYCD3;3 + YFP^N^-GUS were used as negative control.YFP: yellow fluorescent signal; NLS-mCherry: nuclear signal localisation label; Bright field: brightfield vision; Merge: superposition of fluorescence signals, bar = 50 μm.

Previous in vitro yeast two-hybrid (Y2H) experiments and molecular docking experiments showed that PtoCYCD3;3 protein interacts with 12 PtoCDK proteins, of which the strongest interaction is PtoCDKE;2^12^. To verify the reliability of the in vitro Y2H results, in vivo validation in plants was performed using the Bimolecular Fluorescent Complimentary (BIFC) assay. The 12 PtoCDKs screened by Y2H and PtoCYCD3;3 were fused to the N- and C-termini of YFP to construct fusion vectors (YFP^N^–*PtoCDKs* and YFP^C^–*PtoCYCD3;3*), and transient infection mediated by Agrobacterium in two fusion proteins were co-expressed in the lower epidermal cells of tobacco. If the two proteins interacted, the two fragments of the fluorescent protein will be close to each other in space, complementary to each other and reconstructed into an active and complete fluorescent protein molecule, thereby generating fluorescence. At the same time, GUS was fused to the N-terminus of YFP (YFP^N^–GUS) and co-expressed with YFP^C^–PtoCYCD3;3 as a negative control. The 12 PtoCDKs (PtoCDKA;1, PtoCDKB1;1, PtoCDKB1;2, PtoCDKB2;1, PtoCDKB2;2, PtoCDKC;2, PtoCDKD;1, PtoCDKD;2, PtoCDKE;2, PtoCDKF;1, PtoCDKG;3 and PtoCDKG;4) and PtoCYCD3;3, showed fluorescence in the nuclei of tobacco epidermal cells (Fig. 6). The results indicated that PtoCYCD3;3 also interacted with these 12 PtoCDKs proteins in plants.

### PtoCYCD2;1 interacted with PtoCDKA protein

Our previous research found that transgenic *PtoCYCD2;1* and transgenic *PtoCYCD3;3* poplars have completely opposite phenotypes. Transgenic *PtoCYCD2;1* plants have reduced plant height, curled leaves and thin stems^42^, whereas transgenic *PtoCYCD3;3* poplar plants have increased height, curled leaves, thickened stem and branched in advance^12^. Cloned *PtoCYCD2;1* and *PtoCYCD3;3* genes were combined with the CYCD gene families of *P. tomentosa* and *P. trichocarpa* to construct a phylogenetic tree. Results showed that *PtoCYCD2;1* and *PtoCYCD3;3* belonged to the D2 and D3 subclasses, respectively, and closely related to the *PotomCYCD* gene (Fig. S6).

**Figure 7:**
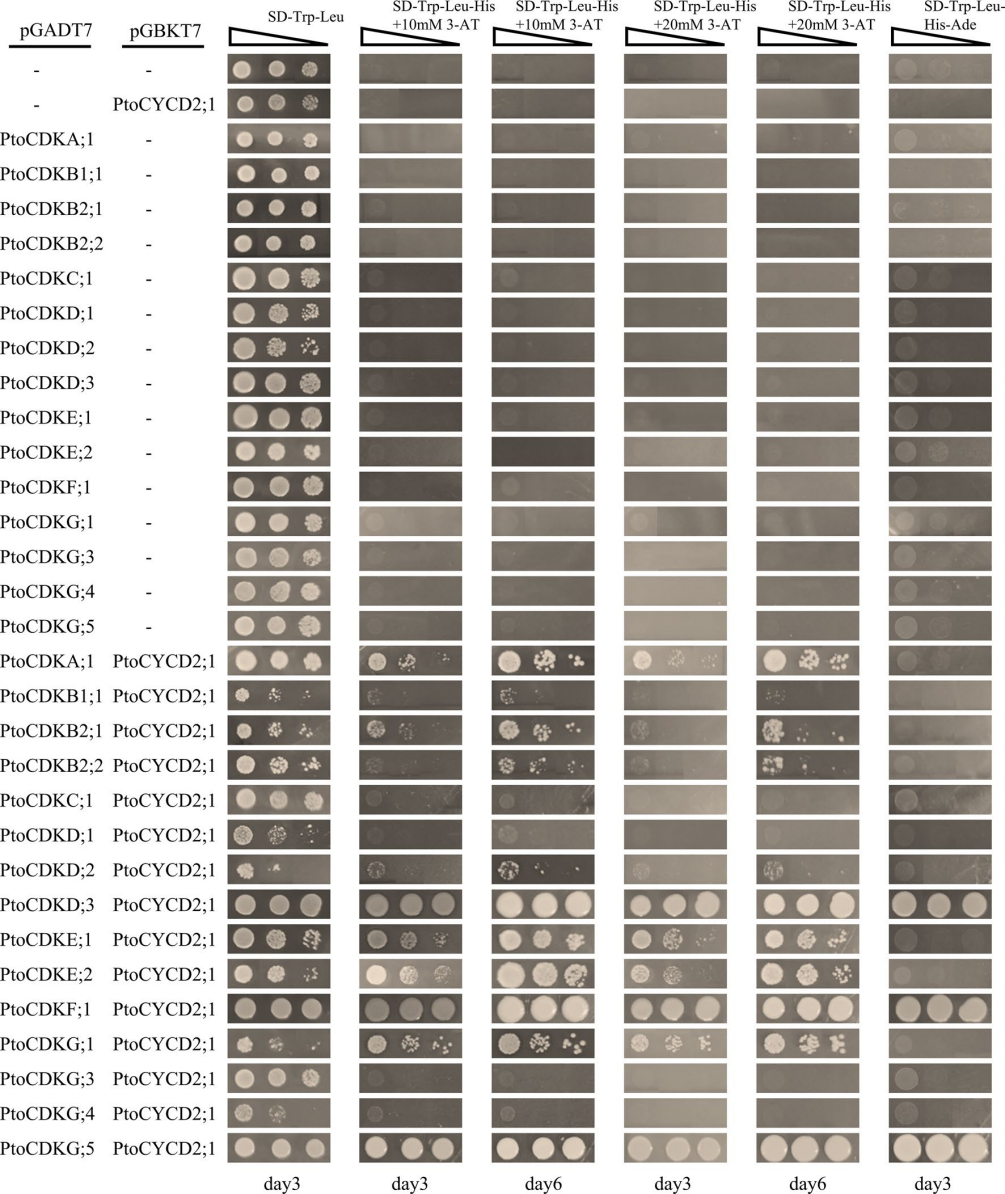
Protein–protein interactions of PtoCYCD2;1 with PtoCDKs. Yeast cells were co-transformed with pGBKT7 and pGADT7 constructs carrying the corresponding genes and grown on SD-Trp-Leu, SD-Leu-Trp-His + 10 mM 3-AT, SD-Leu-Trp-His + 20 mM 3-AT, SD-Leu-Trp-His-Ade. AD + BD, AD + BD-PtoCYCD2;1 and AD-PtoCDKs + BD, were used as negative control. Day represents the number of days of growth on the corresponding medium. The triangle represents dilution in a 0.1-fold gradient (1,0.1 and 0.01).

In order to find out the interaction between PtoCYCD2;1 and PtoCDKs, in vitro Y2H and in vivo BIFC experiments were used to detect the interaction. Y2H vectors (pGBKT7–*PtoCYCD2;1* and pGADT7–*PtoCDKs*) were constructed and co-transformed into yeast AH109 competent cells, and the successfully identified positive yeast strains were spread on different AA-deficient media for Y2H experiment. As a competitive inhibitor of HIS3, 3-AT can inhibit the expression of HIS3 to a certain extent by adding this substance to the culture medium. The growth rates on SD-Trp-Leu-His + 10/20 mM 3-AT and SD-Trp-Leu-His-Ade culture media were observed to detect the interaction strength between PtoCYCD2;1 and different PtoCDKs proteins. During the 6 day observation period, PtoCDKD;3, PtoCDKF;1 and PtoCDKG;5 could grow on SD-Trp-Leu, SD-Trp-Leu-His + 10/20 mM 3-AT and SD-Trp-Leu-His-Ade culture media, suggesting the strongest interaction. PtoCDKA;1, PtoCDKB1;1, PtoCDKB2;1, PtoCDKB2;2, PtoCDKD;2, PtoCDKE;1, PtoCDKE;2 and PtoCDKG;1 could grow on SD-Trp-Leu, SD-Trp-Leu-His + 10/20 mM 3-AT culture media, suggesting a strong interaction. PtoCDKC;1, PtoCDKD;1, PtoCDKG;3 and PtoCDKG;4 could only grow on SD-Trp-Leu culture medium, but not on SD-Trp-Leu-His + 10/20 mM 3-AT and SD-Trp-Leu-His-Ade culture media, indicating that no direct interaction between these proteins (Fig. 7). The growths on the SD-Trp-Leu-His + 10/20 mM 3-AT and SD-Trp-Leu-His-Ade culture media for 3 and 6 days were photographed and observed, and the interaction strength of the PtoCDKs gene family members with PtoCYCD2;1 was observed to have the following relationships: PtoCDKD;3 = PtoCDKF;1 = PtoCDKG;5 > PtoCDKA;1 > PtoCDKG;1 > PtoCDKE;2 > PtoCDKE;1 > PtoCDKB2;1 > PtoCDKB2;2 > PtoCDKD;2 > PtoCDKB1;1.

**Figure 8:**
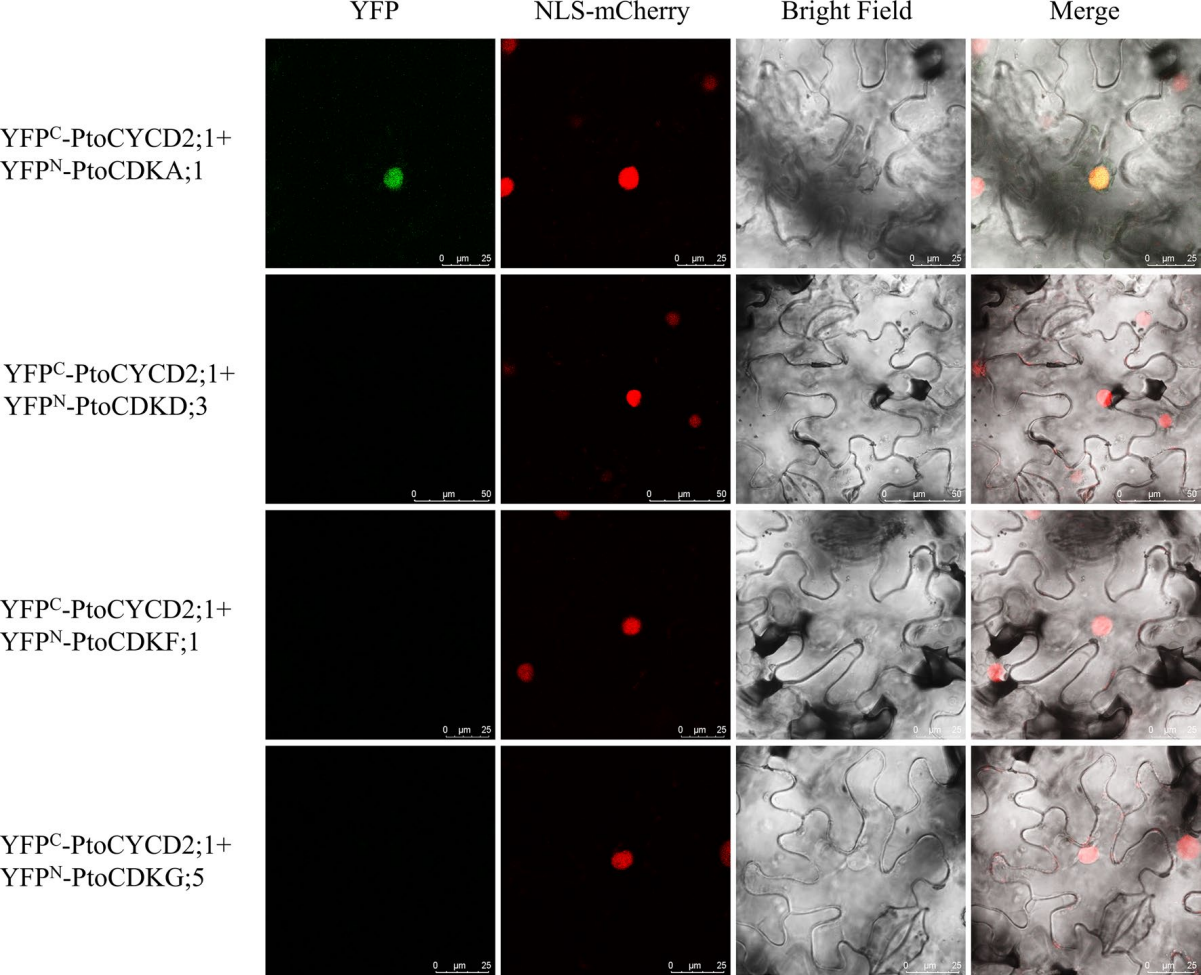
BiFC validation in tobacco epidermis. *PtoCYCD2;1* connect pSPYCE(MR) vector and *PtoCDKs* connect pSPYNE(R)173 vector. After co-expression in tobacco leaf epidermal cells, the fluorescence signal was observed under laser confocal microscopy. YFP: yellow fluorescent signal; NLS-mCherry: nuclear signal localisation label; Bright field: bright field vision; Merge: superposition of fluorescence signals. Scale bars forYFP^C^-PtoCYCD2;1 + YFP^N^-PtoCDKD;3, 50 µm; for others, 25 µm.

To verify the interaction between PtoCYCD2;1 and PtoCDKD;3, PtoCDKF;1, PtoCDKG;5 and the fastest-growing PtoCDKA;1 on SD-Trp-Leu-His + 10/20 mM 3-AT culture medium in plants, we successfully constructed BIFC vectors (YFP^N^–*PtoCDKs* and YFP^C^–*PtoCYCD2;1*), which were transiently expressed in the lower epidermis of tobacco through Agrobacterium-mediated transient infection. Results showed that only PtoCDKA;1 and PtoCYCD2;1 showed fluorescence in the nuclei of tobacco cells and that PtoCDKD;3, PtoCDKF;1, PtoCDKG;5 and PtoCYCD2;1 co-expressed no fluorescence signal (Fig. 8). These results indicated that PtoCYCD2;1 only interacted with PtoCDKA;1 in plants.

## Discussion

In eukaryotes, cyclin-dependent kinases (CDKs) govern the plant cell cycle. It is generally believed that D-type Cyclin (CYCD) can form complexes with CDKs and control the cell cycle G1/S transition^1^. Although the functions of *CYCD* and *CDK* genes have been studied in many species, their functions and evolutionary relationships in some allopolyploid plants and the connection between the two gene families remain unclear. In this study, 43 *CYCDs* and 27 *CDKs* members were identified in *P. tomentosa* (Table S1). Phylogenetic analysis indicates that 43 *CYCD* genes are divided into six subclasses (i.e. D1, D2/4, D3, D4, D5, D6 and D7), of which D1 is the most numerous subclass (12 Members). A total of 27 *CDK* genes are divided into seven subclasses (i.e. CDKA–CDKG), with CDKG being the largest subclass comprising 9 members (Fig. 1). For conserved domains, our study indicates that the most CYCDs contain Cyclin_N and Cyclin_C domains, and PEST motifs are considered responsible for the rapid degradation of these and other unstable proteins^34^. Most CYCDs contain PEST motifs, but PEST positions are not fixed. LxCxE is a key motif for CYCD binding to RBR^44^. No LxCxE motif is found only in the D6 subclass, and LxCxE motifs are present in other subclasses of proteins. The members of the maize D6 subclass also lack the LxCxE structure^45^. This finding may suggest that the D6 subclass plays a special function. Gene structure and motif analyses results indicate that sequences within the same subclass in the *CYCD* and *CDK* gene families are highly similar (Fig. 2). However, different gene structures and conserved motifs existed in some specific alleles which might lead to different functions. The numbers of *CYCD* and *CDK* genes in *P. tomentosa* are significantly more than those in *P. trichocarpa*^12^, and the collinearity analysis of these *CYCD* and *CDK* genes indicates that the expansion of these two gene families is WGD (Table S6) because the 19 pairs of chromosomes in *P. tomentosa* come from different parents^41^. Sequence identity results show that these allele sequences from different subgenomes have some differences (Table S2–S3). Tissue-specific expression analysis shows that these two gene families are more expressed in stems and roots than in leaves. The expression levels of D3 and D1 subclass members in the *CYCD* gene family are highest, followed by that of the D2 subclass (Fig. S4). This finding hints at the importance of members of the three subclasses in cell division and differentiation. In the *CDK* gene family, 2 members of the CDKA family have the highest expression levels in three organs followed by CDKG subclasses. Some CDKG family members have higher expression in leaves than in stems and roots (Fig. S5). This result suggests that CDKA plays an indispensable role in plant growth and development. The CDKG subclass has only 2 members in the herb *Arabidopsis thaliana*^9^, 5 members in *P. trichocarpa*^12^ and 9 members in *P. tomentosa*, and this subclass has the largest number amongst woody plants of CDK subclasses, indicating that CDKG is irreplaceable in some functions. Tissue-specific expression results also show that alleles from different subgenomes are expressed, indicating that they are performing functions at the same time. The predictive analysis of cis-acting elements associated with hormone response and specific expression reveals that *CYCD* and *CDK* gene families are similar, suggesting their functional relatedness.

The interaction between CYCDs and CDKs indicates that the interactions between different CYCD subclasses and CDKs are different. The interactions amongst D1, D2/4, D3, D5, D6, D7 and CDKs are significantly different (Fig. 5), indicating that the functional differences amongst different CYCD members are reflected in evolutionary relationships and sequence characteristics and may be related to the different interactions between them and CDKs. The cyclin interaction network study in *Arabidopsis* shows that the interaction relationship of different cyclins in the protein interaction network is different, which may indicate that the difference in the interaction relationship is closely related to the difference in protein function^46–48^. The D1 subclass CYCD interacts with CDKA, CDKD and CDKE subclasses, and D1 subclass genes are highly expressed in different tissues, indicating the importance of D1 subclass genes in plant development. *Antma;CycD1;1* (CycD1) can promote both G0/G1/S and S/G2/M progression^49^. The overexpression of *PsnCYCD1;1* generates curved leaves and twisted inflorescence.

stems in *Arabidopsis*^50^. The D5 subclass CYCD interacts with five classes of CDKs (i.e. CDKA, CDKB, CDKD, CDKE and CDKF). *Arabidopsis CYCD5;1* is shown to be a quantitative trait gene that controls plant endonuclear replication^51^. The D6 subclass interacts with CDKA, CDKB and CDKD. Maize (*Zea mays L.*) CDKA;1 and CYCD6;1 are shown to interact by in vitro pull-down experiments and have kinase activity^52^. CYCD6;1 has a very specific role in patterning and growth in *Arabidopsis*^53^. Like the D2 subclass, the CYCD7 subclass interacts only with CDKA. Tissue-specific expression indicates that neither D7 subclass genes are expressed. *Arabidopsis CYCD7;1*, neither expressed during seed development, whereas overexpression of *CYCD7;1* promotes the overgrowth of embryo and endosperm through increased division and cell enlargement^54^. The activation of *CYCD7* in the central cell and early endosperm promotes early endosperm and embryo development^39^. The analysis of cis-acting elements also shows that D7 subclass genes contained an endosperm-specific negative expression element. This result suggests that the D7 subclass may play an important role during seed development.

A number of studies showed that plant CYCD2 and CYCD3 have remarkable differences in the regulation of plant growth and development. The ectopic expression of *Triae;CYCD2;1* in *Arabidopsis* affects plant morphology and retards plant growth^55^. AtCYCD2;1 interacts with CDKA;1 only in the presence of ICK2/KRP2, and the activity of CYCD2;1 is closely related to the ICK2/KRP2 inhibition of lateral root branching^37^. The expression of genomic *AtCYCD2;1* in *Arabidopsis* induces cell division at small cell sizes at the root tip^56^. Transgenic *PtoCYCD2;1* makes poplar leaves small and curled, decreases plant height and causes thin stem diameter^42^. In this study, the protein interaction prediction results show that the D2/4 subclass only interact with CDKA (Fig. 5), and this finding is also verified by Y2H and BIFC experiments (Figs. 7 and 8). The overexpression of *Nicta;CycD3;4* genes in tobacco accelerates growth rate^57^. Three *CYCD3* genes of *A. thaliana* are all expressed to be related to the secondary growth of plants^38,58^. A triple loss-of-function *AtCYCD3* mutant stems and hypocotyls show a marked reduction in diameter linked to reduced mitotic activity in the cambium^58^. Branching and axillary shoots are reduced in floral shoots in *AtCYCD3* deletion mutants^59^. The downstream regulatory genes of *AtCYCD3;1* and *E2F* are studied and show a common and distinct function of *CYCD3;1* and *E2F*^60^. Triploid Chinese white poplar (*P. tomentosa Carr., Salicaceae*) has stronger advantages in growth and better stress resistance and wood quality than diploid *P. tomentosa*. The expression levels of *CYCD3* in triploid *P. tomentosa* are higher than those in diploid *P. tomentosa*^61^. Transgenic *PtoCYCD3;3* makes poplar leaves large and curled, increases plant height and stem diameter^12^. The protein interaction prediction results of this study show that the D3 subclass interacts with CDKA and CDKE. Y2H and BIFC experiments confirm this result^12^, and found that PtoCYCD3;3 also interacts with five other subclass CDK proteins (i.e. CDKB, CDKC, CDKD, CDKF and CDKG; Fig. 6).

Plant growth and development and organ formation require cell division and differentiation. In this study, based on the previous research results and literature of the research group, a hypothetical conjecture is put forward due to the phenotypic difference between transgenic *CYCD2* and *CYCD3* plants. Transgenic *PtoCYCD2;1* and *PtoCYCD3;3* poplars produced opposite phenotypes, such as in biomass, plant height, and stem diameter^12,42^(2020,2022). Taking leaf development as an example, in the early stage of leaf growth, the increase in leaf area is predominantly caused by the increase in the number of cells caused by cell division. Afterwards, the rate of cell division slows down, and the increase in leaf area is caused by the increase in cell number and cell enlargement. In the later stages of leaf development, the increase in leaf area is predominantly caused by cell enlargement. Afterward, the area of the leaf does not increase, and some cells in the leaf are still undergoing DNA replication but not cell division, resulting in the appearance of polyploid cells. Stomata are final divisions that occur at specific locations, signaling the end of proliferative activity^62^. This result indicates that cell division capacity in leaf cells is continuously decreasing^62,63^. Transgenic *CYCD2* and *CYCD3* are different in leaves, which may be because the interaction of CYCD2 and CYCD3 with CDKA promotes G1/S transition. This phenomenon promotes cell division. Thus, leaf cells cannot form normal cell shapes to support leaf morphogenesis, ultimately resulting in the production of many small leaf cells and leaf curling. However, transgenic *CYCD2* plants stop cell proliferation at the end of cell proliferation or when a certain number of leaf cells are produced, whereas *CYCD3* transgenic plants can also interact with several other subclasses of CDKs to prolong the time of cell proliferation and increase the leaf area. The stem, as a part where new xylem and phloem cells can be continuously differentiated from cambium cells, is the main display part of cell differentiation ability. The cambium cell thickness is closely related to cambium cell activity^64^. Transgenic *CYCD2;1* plants exit cell proliferation early during primary growth and produce few cambium cells, resulting in a decrease in cambium cell thickness, a decrease in the ability of cambium cells to differentiate into xylem and phloem, and a decrease in stem diameter. This result indicates that the transgenic *CYCD2* inhibits the differentiation of cambium cells into xylem and phloem cells, whereas transgenic *CYCD3* prolongs the time of cell proliferation during primary growth, resulting in increased cambium cell thickness and stem diameter. Thus, transgenic *CYCD3* can promote cambium cells to xylem and phloem cell differentiation. Such different phenotypic changes in transgenic *PtoCYCD2;1* and *PtoCYCD3;3* plants may be due to their different interactions with CDK proteins.

In this study, the *CYCD* and *CDK* genes of allodiploid *P. tomentosa* are identified, and their physicochemical properties, gene structure, chromosome distribution, collinearity and interactions are systematically and comprehensively studied to lay the foundation for future studies of woody plant cell proliferation and differentiation. The relationship between the difference of interaction between CYCD2 and CYCD3 subclasses and CDK protein and the growth and development of woody plants are further analysed. These results provide a new idea and direction for the study of the functions of CYCD and CDK. For perennial woody plants, cell division and differentiation play a crucial role in the entire life cycle process, especially for tree wood production and breeding of high-yielding plants. Based on the previous research results of transgenic phenotypes, this study puts forward a reasonable conjecture to determine the molecular mechanism of cell proliferation-related genes regulating tree growth and development, thus providing certain help for high-yield plant breeding of woody plants.

## Materials and methods

### Database search and phylogenetic analysis for CYCD and CDK members in *P. tomentosa*

*P. tomentosa Carr.* genome (Taxonomy ID: 118,781) was downloaded from the NCBI website. *P. tomentosa CYCD* and *CDK* gene family members were identified using the Hmmer software, NCBI-Blast software and CDD website (https://www.ncbi.nlm.nih.gov/Structure/cdd/wrpsb.cgi). A phylogenetic tree was constructed for classification with PtrCYCD and PtrCDK gene families. The Mafft website (https://www.ebi.ac.uk/Tools/msa/mafft/) was used to construct multiple sequence alignment files. The modelgenenrator software determined the best model^65^, and the phyml-3.1 software built the phylogenetic tree (bootstrap: 1000)^66^. The iTOL website (https://itol.embl.de/) performed phylogenetic tree beautification.

### Sequence analysis

Multiple sequence alignments were performed using PtrCDKs and PotomCDKs protein sequences through the BioEdit tool. The ProtParam website (https://web.expasy.org/protparam/) was used to predict protein physicochemical properties, and the Plant-mPLoc website (http://www.csbio.sjtu.edu.cn/bioinf/plant-multi/) was used to predict protein subcellular localisation. Cyclin_N and Cyclin_C domains were predicted using the CDD website (https://www.ncbi.nlm.nih.gov/Structure/cdd/wrpsb.cgi). The epestfind website (http://emboss.bioinformatics.nl/cgi-bin/emboss/epestfind) predicted the PEST domain of the protein, and the score threshold was set to 0. LxCxE motifs were predicted using the website (http://elm.eu.org/elms/LIG_Rb_LxCxE_1). The sequence identity analysis was performed using the BioEdit tool. The exon/intron organisation for each individual *PotomCYCDs* and *PotomCDKs* was displayed using GSDS online website (http://gsds.gao-lab.org/). Using the MEME website (https://meme-suite.org/meme/tools/meme) predict conservative motif, parameter setting: Zero or one occurrence oer sequence (zoops), select the number of motifs: 10, minimum width: 6 and maximum width: 256/133 (the shortest protein length in the protein sequence of PotomCYCDs and PotomCDKs protein). Cis-acting elements were predicted in the 2000 bp upstream regions by using the PlantCARE website (http://bioinformatics.psb.ugent.be/webtools/plantcare/html/). Cis-acting elements were visualised uses the TBtools v1.098691^67^.

### Chromosomal distribution and synteny analyses

The genomes of *P. trichocarpa* and *A. thaliana* were obtained from the Phytozome (https://phytozome-next.jgi.doe.gov/) and TAIR (https://www.arabidopsis.org/), respectively. Detailed information of chromosomal distribution was acquired from the *P. tomentosa* genome database. All *CYCD* and *CDK* genes of *P. tomentosa* were mapped onto chromosomes by TBtools. The synteny analysis and distinct duplication events were calculated via MCScanX^68^. The synteny relationships of *CYCD* and *CDK* genes with different genomes were exhibited using TBtools.

### Transcriptome data acquisition and heatmap drawing

The original transcriptome data leaf (SRR11321035), stem (SRR11321036), root (SRR11321037) were downloaded from NCBI. The Fastp software was used for the quality control of the original data, and the hisat2 software was used for genome comparison. The comparison rates were as follows: leaf, 98.15%; stem, 97.39%; root, 97.52%. The HTseq software was used to calculate the gene expression matrix^69^. The gene length was counted using the GenomicFeatures software, and the FPKM values of all genes were calculated at the same time^70^. After extracting the FPKM of the gene, the log2(FPKM + 1) was calculated and the TBtools software was used for heatmap drawing.

### Prediction of protein–protein interaction

The interaction between *PotomCYCDs* and *PotomCDKs* gene family members was predicted using the STRING website (https://cn.string-db.org/) with the following parameters: organism, *A. thaliana*; active interaction sources, experiments.

### BIFC experiments

The coding sequences of *PtoCDKs* were cloned into the pSPYNE(R)173 vector. The coding sequences of *PtoCYCD2;1* and *PtoCYCD3;3* were cloned into the pSPYCE(MR) vector^71^. The constructed plasmids were transformed into *Agrobacterium* GV3101 competent cells middle. Positive strains were selected and mixed to infect tobacco leaves. After culturing for 48 h, leaves were cut and observed using the Leica TCS SP8 confocal laser scanning platform(Beijing Forestry University) to observe the fluorescence signal. The excitation light wavelength was set to 488 nm. The excitation wavelength of NLS-mCherry was set to 587 nm. Primer sequences are listed in Table S9.

### Y2H assays

The coding sequences of *PtoCDKs* were cloned into the pGADT7 vector. The coding sequence of *PtoCYCD2;1* was cloned into the pGBKT7 vector. Successfully sequenced recombinant vectors were co-transformed into yeast strain AH109 competent cells by using the PEG/LiAc method. The kit media used in Y2H experiments were purchased from Coolaber, China. Primer sequences are listed in Table S9.

## Supplementary Information

Supplementary Information.

## Data Availability

The raw sequence datasets generated and analyzed during the current study are available through NCBI datasets (https://www.ncbi.nlm.nih.gov/data-hub/taxonomy/118781/). The gene expression data analyzed during the current study are available at SRA database with accession numbers: SRR11321035, SRR11321036 and SRR11321037. The sequence information of genes from *P. tomentosa* described in this article were deposited into GenBank. Below are the accession numbers of the *P. tomentosa* genes, PtoCDKA;1 (MT990424), PtoCDKB1;1 (MT990425), PtoCDKB1;2 (MT990426), PtoCDKB2;1 (MT990427), PtoCDKB2;2 (MT990428), PtoCDKC;1 (MT990429), PtoCDKC;2 (MT990430), PtoCDKD;1 (MT990431), PtoCDKD;2 (MT990432), PtoCDKD;3 (MT990433), PtoCDKE;1 (MT990434), PtoCDKE;2 (MT990435), PtoCDKF;1 (MT990436), PtoCDKG;1 (MT990437), PtoCDKG;3 (MT990438), PtoCDKG;4 (MT990439), PtoCDKG;5 (MT990440), PtoCYCD3;3 (MT990443) and PtoCYCD2;1 (ON583825). The datasets supporting the conclusions of this article are included in the article and its supplementary materials.
