# Supplementary material for: Study on the interaction preference between CYCD subclass and CDK family members at the poplar genome level
Source: Sci Rep. 2022 Oct 7;12:16805. doi: 10.1038/s41598-022-20800-9 (PMC9547009; doi:10.1038/s41598-022-20800-9)
Supplement: Supplementary file 1 — Supplementary Information. [file 41598_2022_20800_MOESM1_ESM.zip › Supplementary materials/Fig. S4. Heatmap of PotomCYCDs in Different Tissues.pdf]

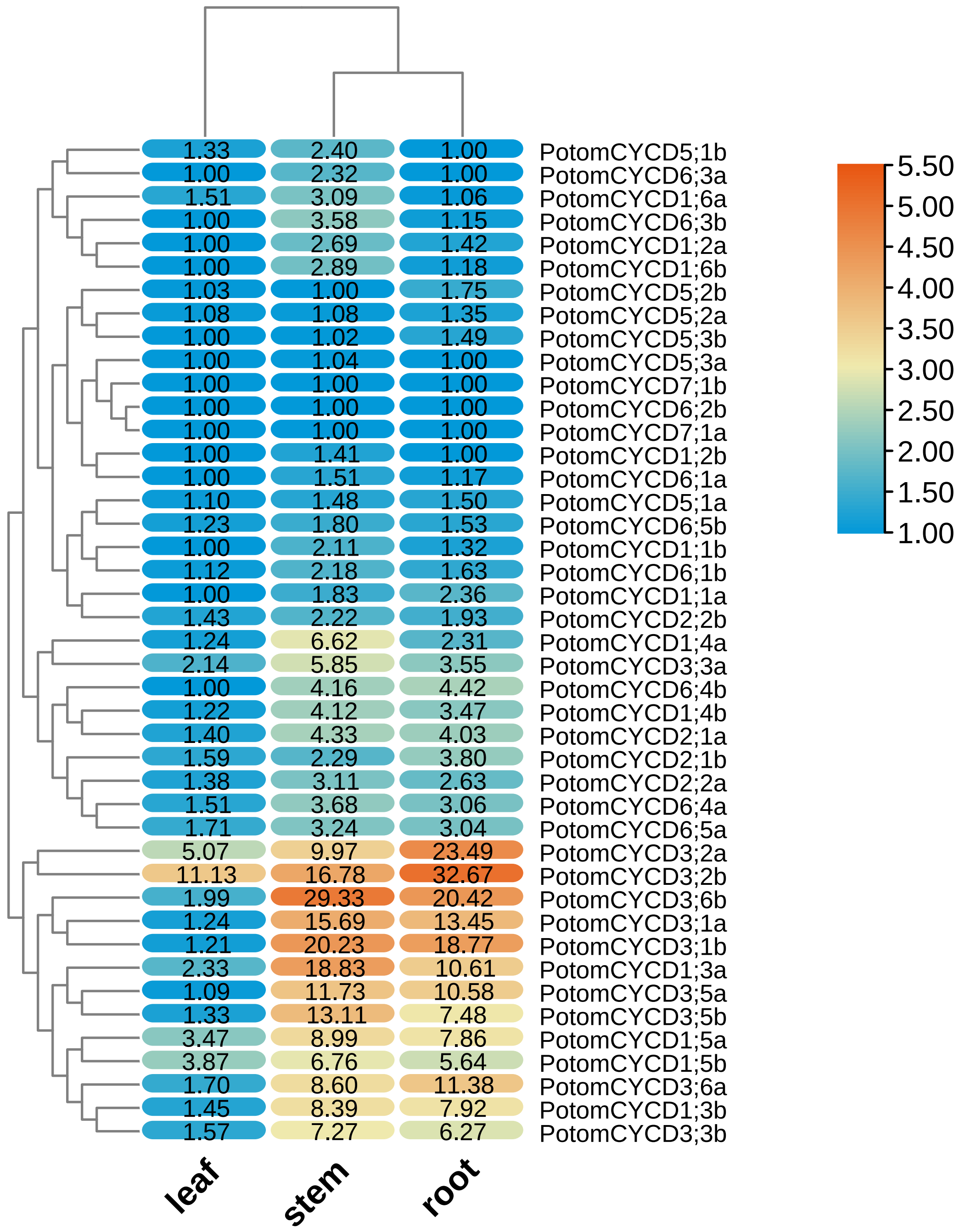
leaf
stem
root
1.33 2.40 1.00 PotomCYCD5;1b
1.00 2.32 1.00 PotomCYCD6;3a
1.51 3.09 1.06 PotomCYCD1;6a
1.00 3.58 1.15 PotomCYCD6;3b
1.00 2.69 1.42 PotomCYCD1;2a
1.00 2.89 1.18 PotomCYCD1;6b
1.03 1.00 1.75 PotomCYCD5;2b
1.08 1.08 1.35 PotomCYCD5;2a
1.00 1.02 1.49 PotomCYCD5;3b
1.00 1.04 1.00 PotomCYCD5;3a
1.00 1.00 1.00 PotomCYCD7;1b
1.00 1.00 1.00 PotomCYCD6;2b
1.00 1.00 1.00 PotomCYCD7;1a
1.00 1.41 1.00 PotomCYCD1;2b
1.00 1.51 1.17 PotomCYCD6;1a
1.10 1.48 1.50 PotomCYCD5;1a
1.23 1.80 1.53 PotomCYCD6;5b
1.00 2.11 1.32 PotomCYCD1;1b
1.12 2.18 1.63 PotomCYCD6;1b
1.00 1.83 2.36 PotomCYCD1;1a
1.43 2.22 1.93 PotomCYCD2;2b
1.24 6.62 2.31 PotomCYCD1;4a
2.14 5.85 3.55 PotomCYCD3;3a
1.00 4.16 4.42 PotomCYCD6;4b
1.22 4.12 3.47 PotomCYCD1;4b
1.40 4.33 4.03 PotomCYCD2;1a
1.59 2.29 3.80 PotomCYCD2;1b
1.38 3.11 2.63 PotomCYCD2;2a
1.51 3.68 3.06 PotomCYCD6;4a
1.71 3.24 3.04 PotomCYCD6;5a
5.07 9.97 23.49 PotomCYCD3;2a
11.13 16.78 32.67 PotomCYCD3;2b
1.99 29.33 20.42 PotomCYCD3;6b
1.24 15.69 13.45 PotomCYCD3;1a
1.21 20.23 18.77 PotomCYCD3;1b
2.33 18.83 10.61 PotomCYCD1;3a
1.09 11.73 10.58 PotomCYCD3;5a
1.33 13.11 7.48 PotomCYCD3;5b
3.47 8.99 7.86 PotomCYCD1;5a
3.87 6.76 5.64 PotomCYCD1;5b
1.70 8.60 11.38 PotomCYCD3;6a
1.45 8.39 7.92 PotomCYCD1;3b
1.57 7.27 6.27 PotomCYCD3;3b
5.50
5.00
4.50
4.00
3.50
3.00
2.50
2.00
1.50
1.00
