# Supplementary material for: Study on the interaction preference between CYCD subclass and CDK family members at the poplar genome level
Source: Sci Rep. 2022 Oct 7;12:16805. doi: 10.1038/s41598-022-20800-9 (PMC9547009; doi:10.1038/s41598-022-20800-9)
Supplement: Supplementary file 1 — Supplementary Information. [file 41598_2022_20800_MOESM1_ESM.zip › Supplementary materials/Supplementary materials description.docx]

**Supplementary Materials: Fig. S1.** Visualization of CYCD family conserved domains. Different colored boxes represent different domains or from converted motifs. **Fig. S2.** Cis-Acting Elements of *PotomCYCD* gene family. a. Statistics on the number of hormone-responsive cis-acting elements. b. Statistics of the number of cis-acting elements related to specific expression. c. Location of hormone response-related cis-acting elements in each *PotomCYCDs*. **Fig. S3.** Cis-Acting Elements of *PotomCDK* gene family. a. Statistics on the number of hormone-responsive cis-acting elements. b. Statistics of the number of cis-acting elements related to specific expression. c. Location of hormone response-related cis-acting elements in each *PotomCDKs.* **Fig. S4.** Heatmap of *PotomCYCDs* in Different Tissues. The log2(FPKM+1) of each gene is the scale. **Fig. S5.** Heatmap of *PotomCDKs* in Different Tissues. The log2(FPKM+1) of each gene is the scale. **Fig. S6.** Phylogenetic tree. *PtoCYCD2;1* and *PtoCYCD3;3* in red boxes were cloned from *Populus tomentosa Carr.*(Clone 741). **File S1.** Sequence alignment of CDK family. **Table S1.** Gene_name and sequence of *PotomCYCDs* and *PotomCDKs*. **Table S2.** PotomCYCD sequence identity. **Table S3.** PotomCDK sequence identity. **Table S4.** Sequence Characteristics of *PotomCYCDs* and *PotomCDKs*. **Table S5.** Motif sequence. **Table S6.** *PotomCYCD* and *PotomCDK* gene pairs. **Table S7.** *CYCD* and *CDK* gene pairs in three species. **Table S8**. Comparison results. **Table S9**. Primers sequence.
