# Supplementary figures and images for: Study on the interaction preference between CYCD subclass and CDK family members at the poplar genome level
Source: Sci Rep. 2022 Oct 7;12:16805. doi: 10.1038/s41598-022-20800-9 (PMC9547009; doi:10.1038/s41598-022-20800-9)

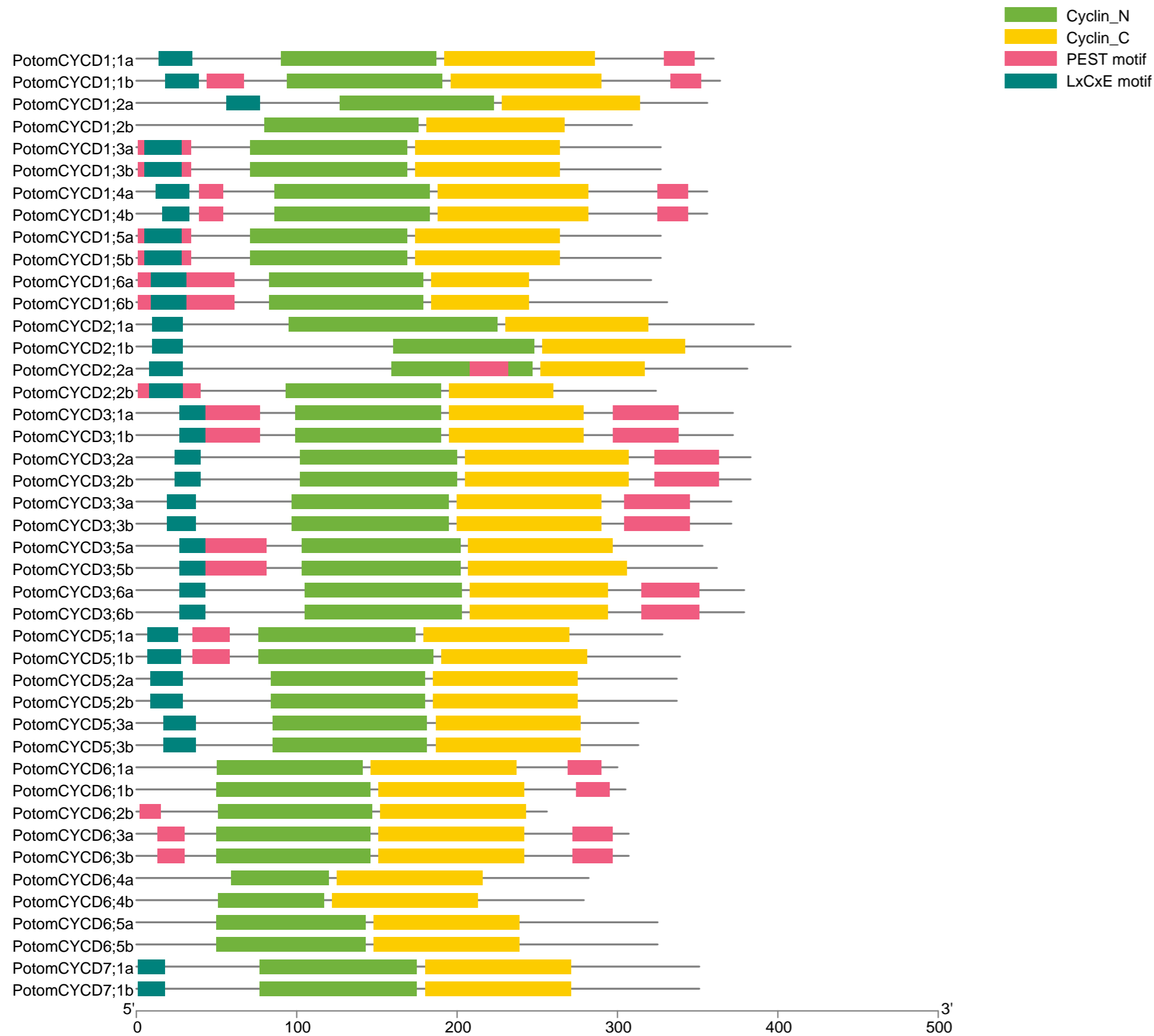

Supplement: Supplementary file 1 — Supplementary Information. [file 41598_2022_20800_MOESM1_ESM.zip › Supplementary materials/Fig. S1. Visualization of CYCD family conserved domains.pdf]

a

## Hormone

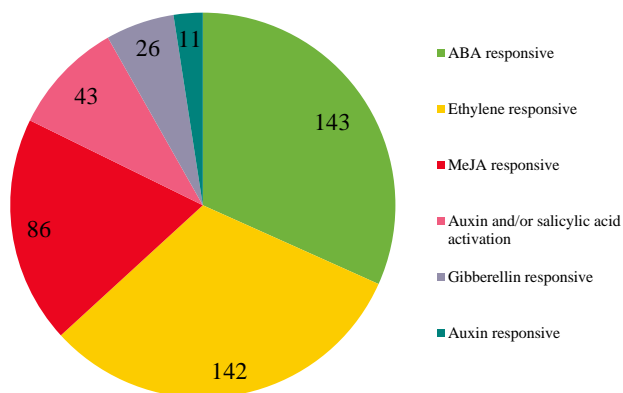

b

## Specific expression

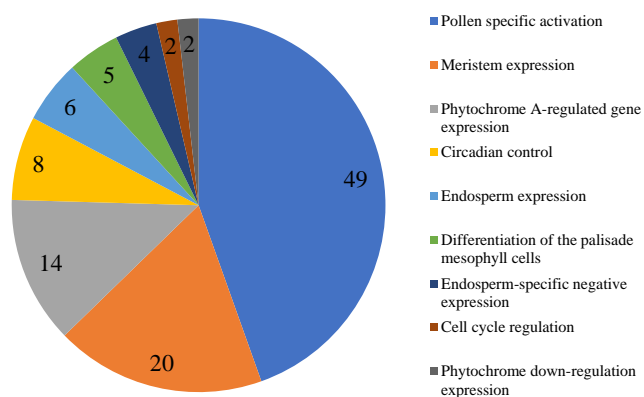

c

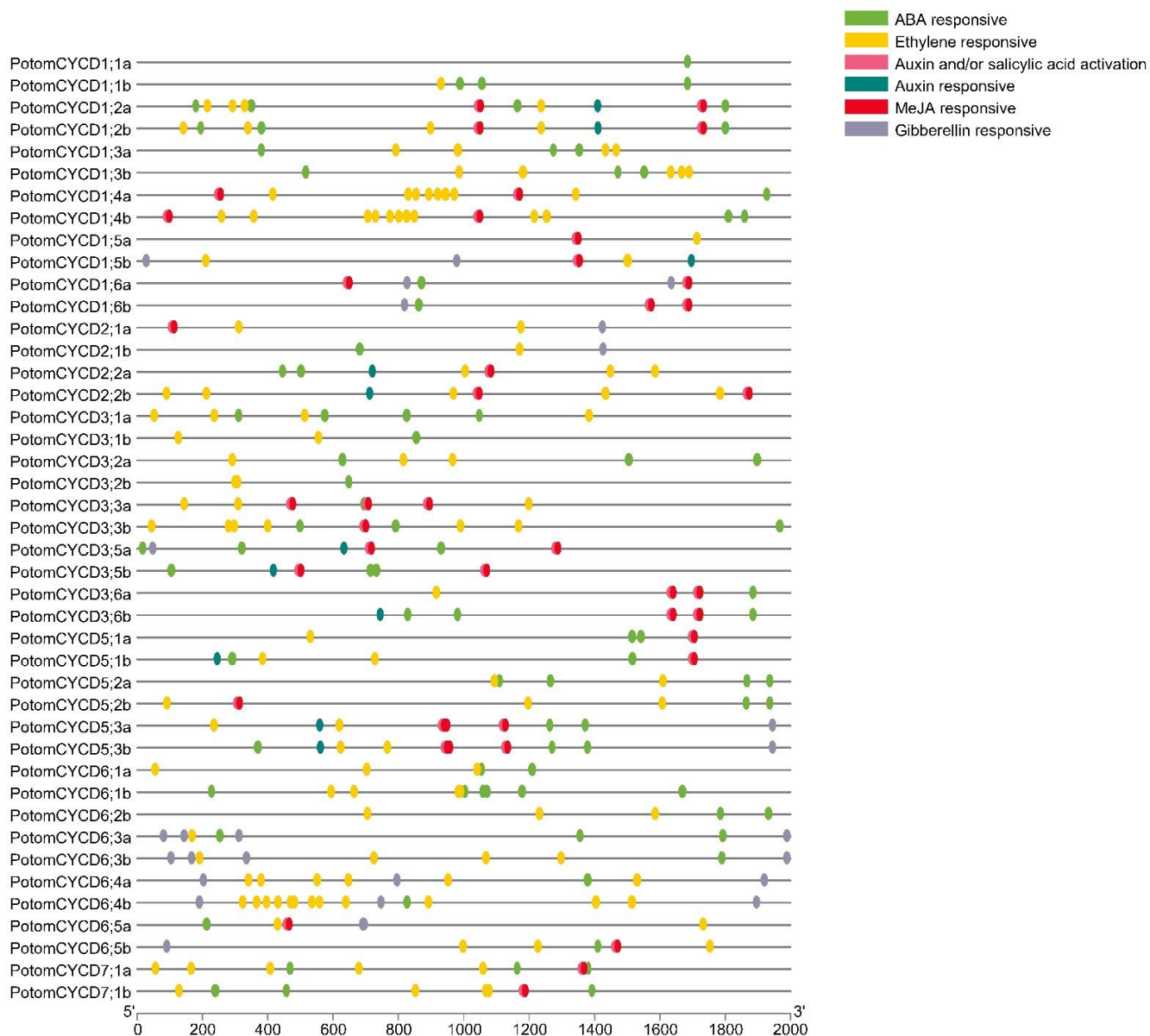

Supplement: Supplementary file 1 — Supplementary Information. [file 41598_2022_20800_MOESM1_ESM.zip › Supplementary materials/Fig. S2. Cis-Acting Elements of PotomCYCD gene family.pdf]

a

## Hormone

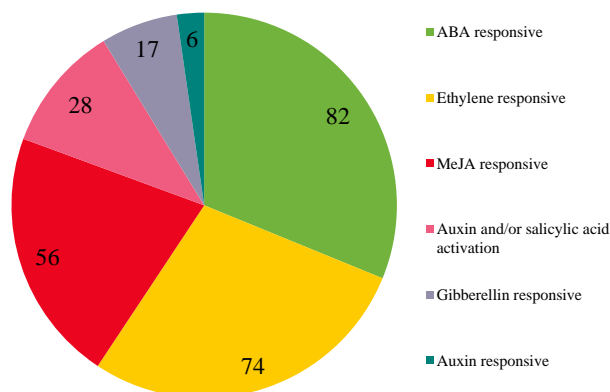

b

## Specific expression

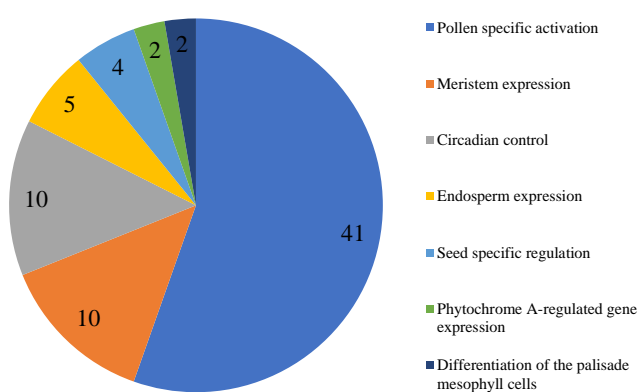

c

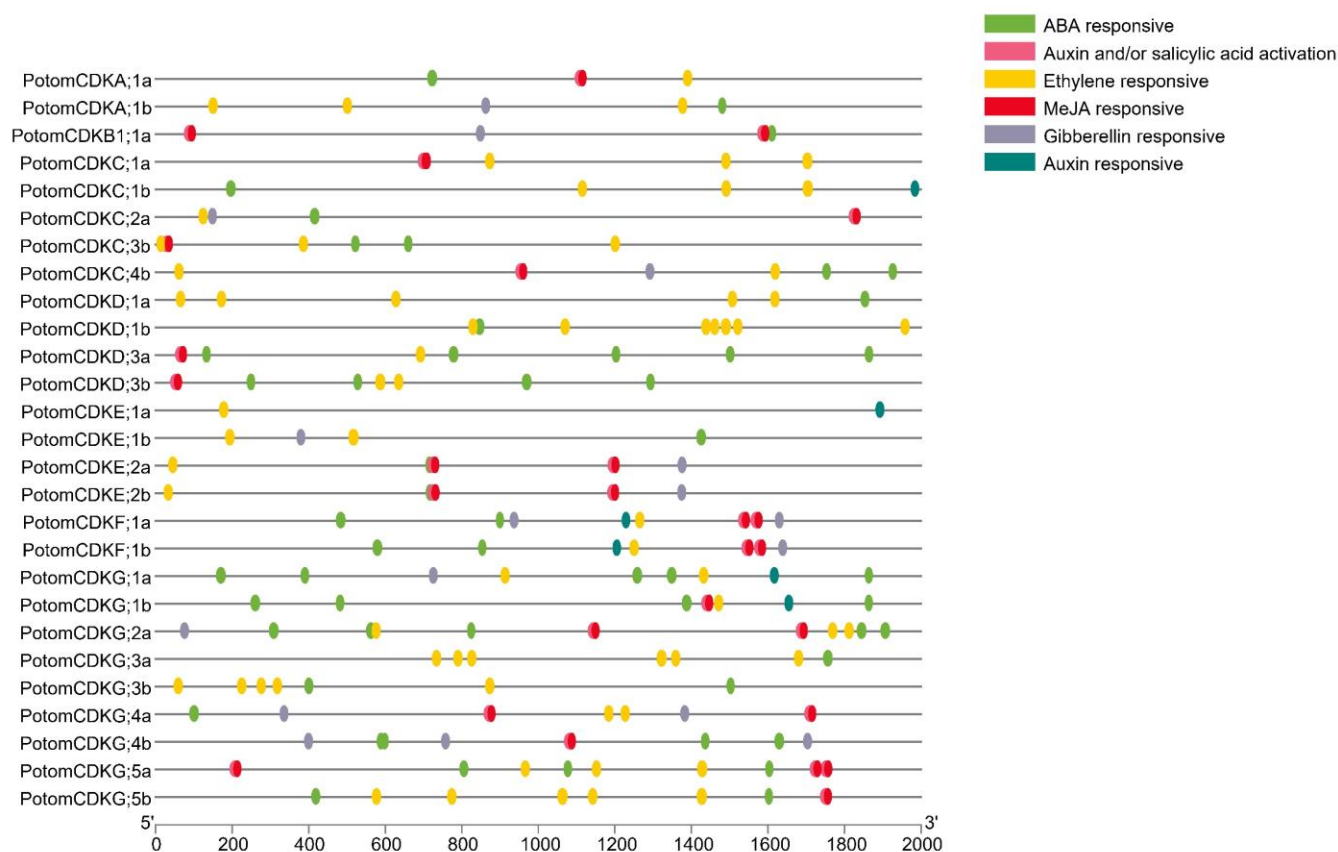

Supplement: Supplementary file 1 — Supplementary Information. [file 41598_2022_20800_MOESM1_ESM.zip › Supplementary materials/Fig. S3. Cis-Acting Elements of PotomCDK gene family.pdf]

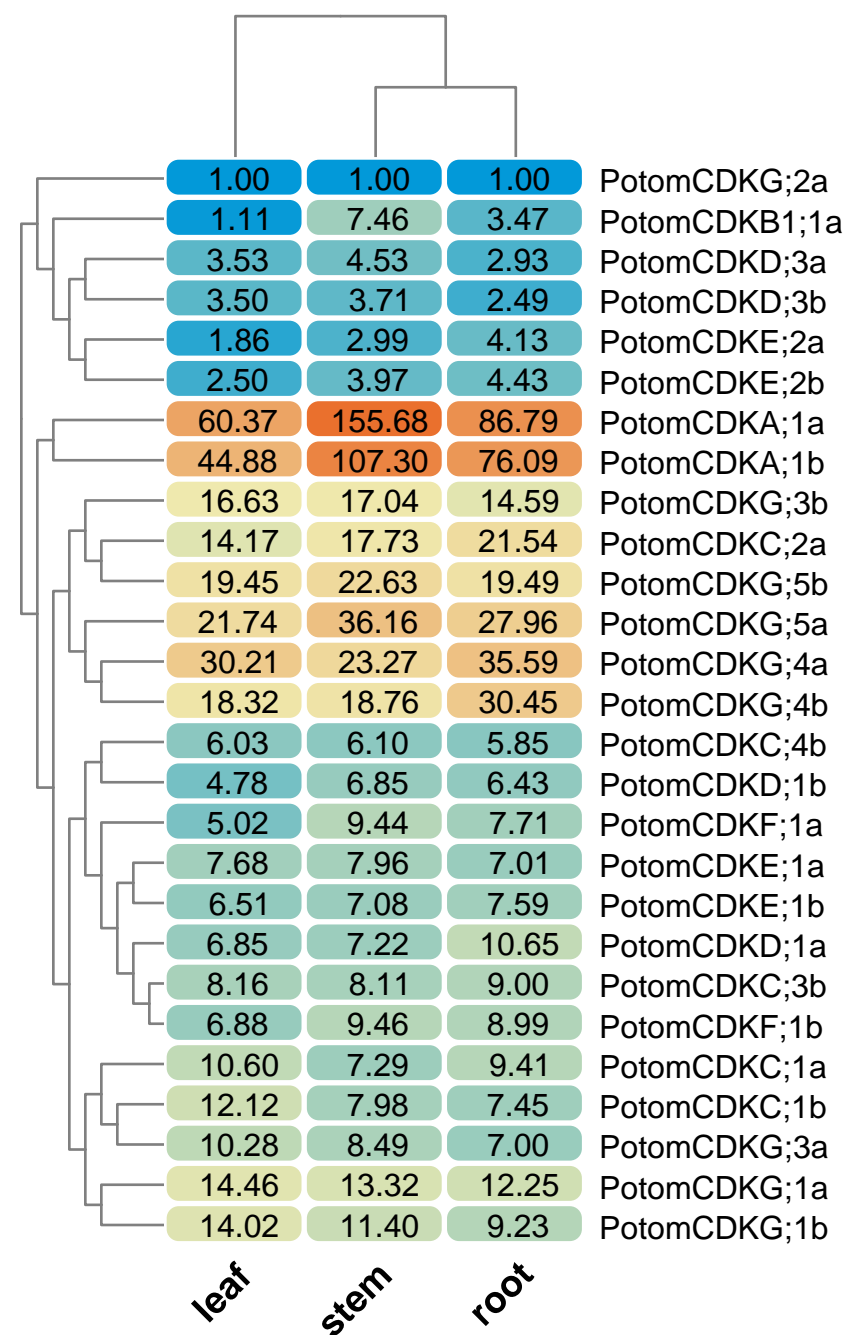

Supplement: Supplementary file 1 — Supplementary Information. [file 41598_2022_20800_MOESM1_ESM.zip › Supplementary materials/Fig. S5. Heatmap of PotomCDKs in Different Tissues.pdf]

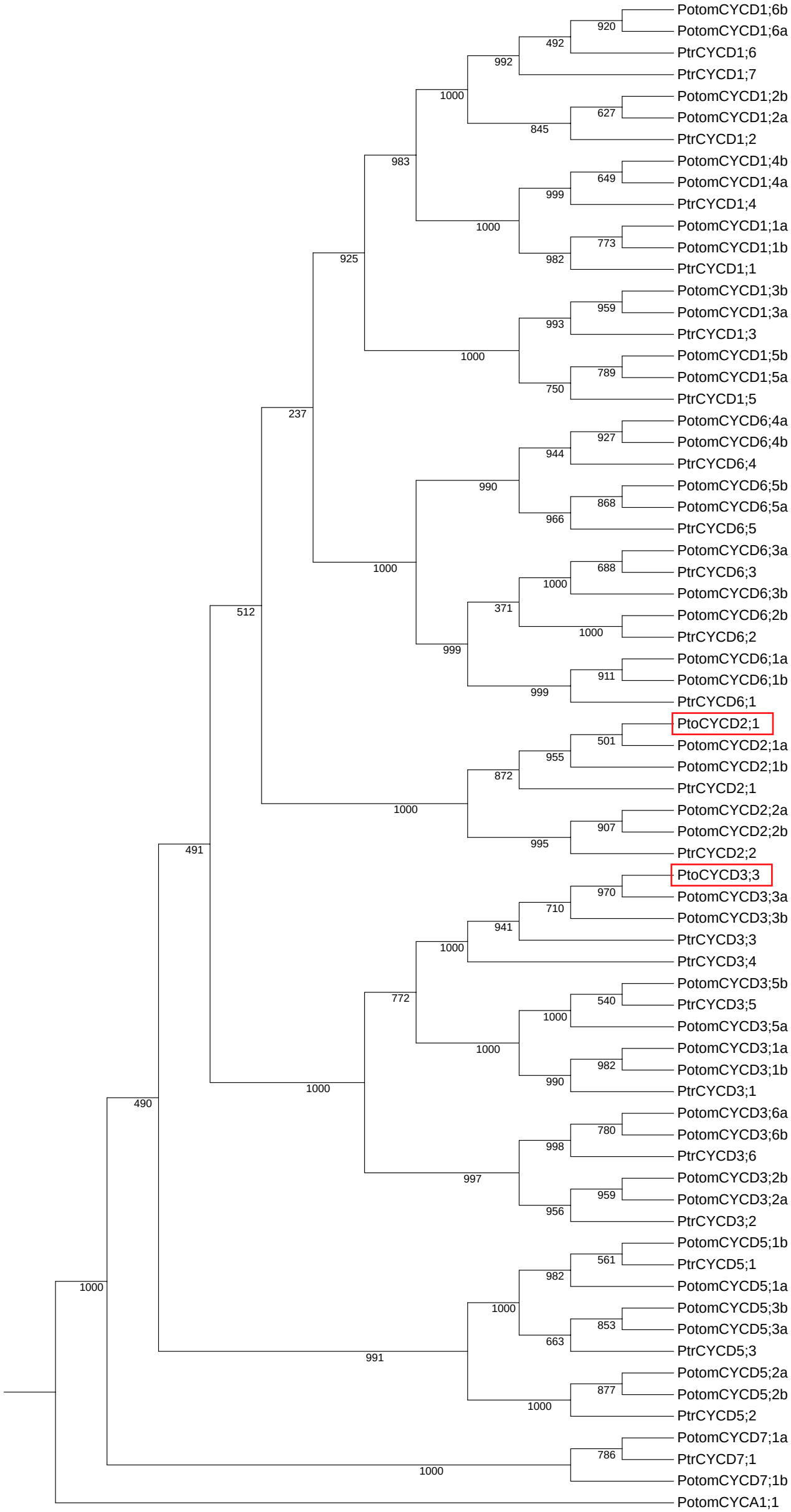

Supplement: Supplementary file 1 — Supplementary Information. [file 41598_2022_20800_MOESM1_ESM.zip › Supplementary materials/Fig.S6. Phylogenetic tree.pdf]

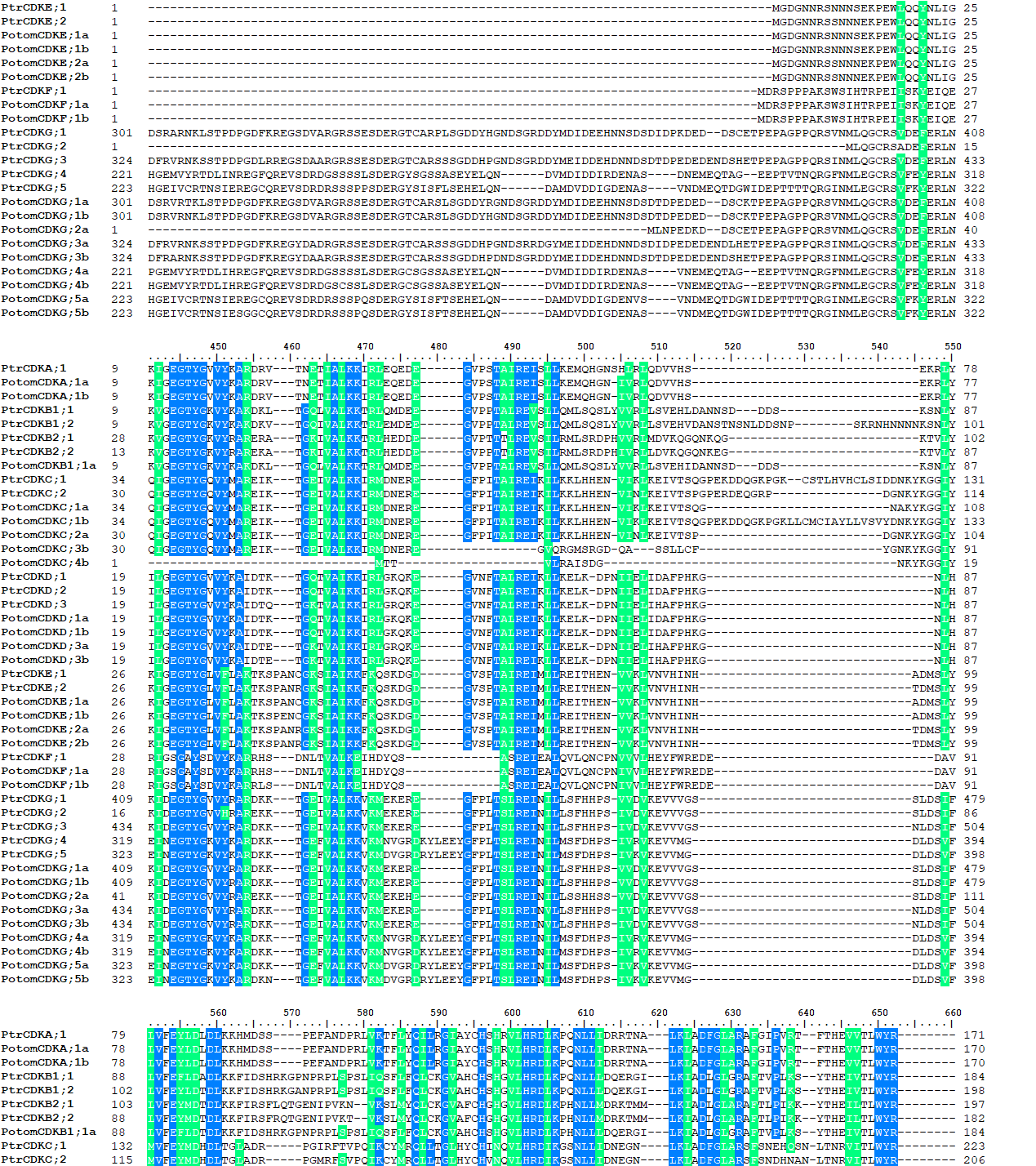


Characteristic motifs

Supplement: Supplementary file 1 — Supplementary Information. [file 41598_2022_20800_MOESM1_ESM.zip › Supplementary materials/File S1. Sequence alignment of CDK family.docx]
